# Ultra‐Low Intensity Continuous Wave Laser Ablation Propulsion With Graphene‐Engineered Wood

**DOI:** 10.1002/advs.75463

**Published:** 2026-05-16

**Authors:** Afnan S. M. Elmubasher, Rami Elkaffas, Mohamed Hamid Salim, Basel Altawil, Chanaka Sandaruwan, Shanavas Shajahan, Ahsan Baidar Bakht, Irfan Hussain, Blaise L. Tardy, Sean S. M. Swei, Yarjan Abdul Samad

**Affiliations:** ^1^ Department of Aerospace Engineering Khalifa University Abu Dhabi UAE; ^2^ Khalifa University Space Technology and Innovation Lab Khalifa University Abu Dhabi UAE; ^3^ Department of Chemical Engineering Khalifa University Abu Dhabi UAE; ^4^ Food Security and Technology Center Khalifa University Abu Dhabi UAE; ^5^ Research and Innovation Centre on 2D Materials Khalifa University Abu Dhabi UAE; ^6^ Department of Mechanical and Nuclear Engineering Khalifa University Abu Dhabi UAE; ^7^ Department of Electrical Engineering University of Cambridge Cambridge UK; ^8^ Advanced Research and Innovation Center (ARIC) Khalifa University Abu Dhabi UAE

**Keywords:** delignification, graphene, laser ablation propulsion, wood

## Abstract

Conventional propellant materials, including polymers, metals, and doped polymers, have long been investigated for laser ablation propulsion, though achieving desired specific impulse values with low‐intensity continuous wave laser irradiation remains a challenge. This study presents low‐density graphene‐delignified wood composites as novel, sustainable propellants for continuous‐wave laser ablation propulsion. Experimental results show that natural wood achieves a density‐specific specific impulse of 5043.0 ± 188 s g^−1^ cm^3^, surpassing the performance of all previously reported conventional propellants. Graphene delignified wood improves the absorption of laser energy of natural wood by 98.61%, thereby achieving an ultra‐low ablation threshold intensity of 0.54 MW m^−2^; the lowest intensity attained so far, and an exceptional density‐specific specific impulse of 1569.60 ± 57.40 s g^−1^ cm^3^. The absolute specific impulse values of NW and GDW are 907.74 and 800.49 s, respectively, whereas no other material has achieved a value >100 s under CW laser irradiation. Additionally, graphene delignified wood has a tensile strength of 273.1 MPa, ∼10 times greater than NW, and a specific tensile strength of 533 MPa g^−1^ cm^3^, exceeding common aerospace structural materials in strength‐to‐weight ratio. The findings unlock the potential of lightweight and sustainable graphene delignified wood for use in various light and space applications.

## Graphical abstract

Graphene delignified wood converts continuous wave laser energy into thrust. Laser propulsion experiments in vacuum reveal the lowest laser ablation threshold intensities and the greatest density‐normalized specific impulse in literature. The results highlight graphene delignified wood as a potential efficient, sustainable, strong, and lightweight propellant material for future micro‐propulsion applications.

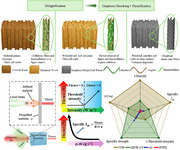

## 1 Introduction

The demand for new spacecraft technology continues to grow in the field of space exploration [1]. Micro/nano satellites contribute to this growth due to their small form factor platform, lighter weight, and flexible launch opportunities [2], increasing their utilization [3, 4] in multiple fields [5]. Micro/nano satellites require micro propulsion systems with thrust magnitudes in the order of µN‐mN to perform orbital maneuvers and attitude adjustments [6]. Laser ablation propulsion (LAP), first introduced by Kantrowitz in 1972, has garnered attention as a promising micro propulsion technology [7]. A laser beam is directed toward a target material, which absorbs the laser energy and undergoes rapid heating, resulting in material evaporation and/or ionization [8]. The expulsion of ablated material from the target surface produces a recoil force or thrust in the opposite direction, hence propelling the target. LAP eliminates the need for traditional pressurized propellant tanks and valves, providing scalability and simplicity best suited for micro/nano satellite missions [9, 10]. The LAP mechanism allows for a wide range of materials to be utilized as propellants, the selection of which is a primary contributor to the performance of the LAP system [11, 12, 13]. LAP propellants are evaluated at two key performance metrics: the impulse coupling coefficient (*C*_m_) and the specific impulse (*I*_sp_) [9]. *C*_m_ denotes the ratio between the thrust generated on the target and the laser input power during ablation, which measures the efficiency of converting laser energy into kinetic momentum, and *I*_sp_ is the ratio between the thrust generated on the target and the rate of mass loss during ablation, which is positively related to the exhaust velocity of the ablated materials.

Currently, LAP propellants include carbon materials [14, 15], metals [11, 15, 16], polymers [17, 18, 19], and metal–organic frameworks (MOFs) [20]. Metals possess high ionization rates and low mass consumption rates, leading to shallow ablation depths and high *I*_sp_ in the range of 10^3^–10^4^ s [21]. However, their high thermal conductivities and boiling points lead to high ablation threshold intensities in the range of 10^1^ ^2^–10^14^ W m^−2^, resulting in low *C*_m_ in the range of 0.1–10 N MW^−1^ [22, 23, 24, 25, 26]. Moreover, metals have the risk of producing spallation particles, which can oxidize in low Earth orbits, forming catalytic sites for ozone depletion [27, 28]. Polymers, with low thermal conductivities and melting and boiling points [29], possess low ablation thresholds in the range of 10^8^–10^1^ ^1^ W m^−2^ and high *C*_m_ in the range of 10–100 N MW^−1^, though their poor energy absorption efficiencies and high mass consumption rates result in large ablation depths [30] and lower *I*_sp_ in the range of 77–600 s. Approaches to enhance the performance of polymers include using engineered energetic polymers [31], and/or carbon and metal dopants [32], but energetic polymers release harmful reactive gases [33], and the non‐uniform dispersion of dopants in polymers leads to their premature ejection, areas of local collapse, and/or agglomeration [34]. Achieving ablation intensity thresholds for those propellants mentioned above requires either high‐power continuous wave (CW) lasers in the range of 10^2^–10^4^ W or ultrashort duration pulsed lasers in the range of 10^−^^1^ ^5^–10^−^^9^ s [35]. However, both are incompatible with current micro/nano satellite platforms. Hence, the ideal LAP propellant would combine the low density and low ablation threshold intensity of polymers with the high strength‐to‐weight ratio and high *I*_sp_ performance of carbon and metals, while ensuring environmental safety. We introduce graphene delignified wood as a candidate material for low‐intensity CW LAP. Wood was selected as a candidate CW‐LAP propellant not only because its low density and sustainability [36, 37] make it attractive for space applications [38], but also for its low thermal conductivity and hierarchical polymeric structure with intrinsic multiscale porosity and anisotropy [39]. As shown in our figures, the cellular architecture of wood provides a lightweight scaffold that can be engineered across multiple length scales, including delignification [40] and the infusion of functional additives such as nanomaterials [41]. Delignification allows control of the chemical composition and microstructure of GDW composites and enables tuning of their LAP performance. Graphene was selected as an additive because it can absorb light at a wide spectrum (UV–vis–NIR) [42], possesses a thermal conductivity of 5300 W m^−1^ K^−1^ [43] and an optical transmittance of ∼97.7% [44, 45], which inspired various studies in photonic propulsion [46, 47, 48, 49, 50, 51]. Graphene has been shown to enhance the light absorption efficiency of LAP propellants [28, 52] leading to lower threshold intensities and higher propulsion forces. The wood framework provides a tunable, porous, and mechanically stable support, while the graphene phase contributes carbon‐rich aromatic structures that are favorable for laser–material interactions. This combination distinguishes the material from conventional dense carbon‐based propellants and from bulk synthetic polymers, while also offering a more structurally robust and scalable platform than highly engineered porous materials such as MOFs. Accordingly, the objective of this work is to investigate how the modified hierarchical structure of wood and the addition of graphene affect laser ablation and propulsion behavior.

The developed GDW combines the low thermal conductivity of wood with the efficient energy absorption of graphene, resulting in the lowest ablation threshold intensity of 0.54 MW m^−2^, making it an attractive LAP propellant. GDW achieved an *I*_sp_ of 800.49 ± 28.51 s, which has never been achieved with CW LAP before. We introduce density‐specific *I*_sp_ = *I*_sp_/ρ, where ρ is the material density, as a complementary propulsion metric to highlight materials that combine high propellant utilization efficiency with low structural mass. The low density (0.51 g cm^−3^) led to a specific *I*_sp_ of 1569.60 ± 57.40 s, 40.14% greater than GMM, with the greatest specific *I*_sp_ value in literature [53]. An attribute of high‐density‐specific *I*_sp_ indicates that for an equal volume of propellant, a smaller mass will be utilized to achieve the total impulse required for a mission. This is particularly advantageous for efficient mass‐budget utilization in the design of micro/nano satellites with stringent mass and volume constraints. Additional GDW properties include formability due to flexibility and a specific tensile strength (533 MPa g^−1^ cm^3^). These unique characteristics of GDW composites make them a leading candidate for next‐generation space materials.

## 2 Results and Discussion

**FIGURE 1:**
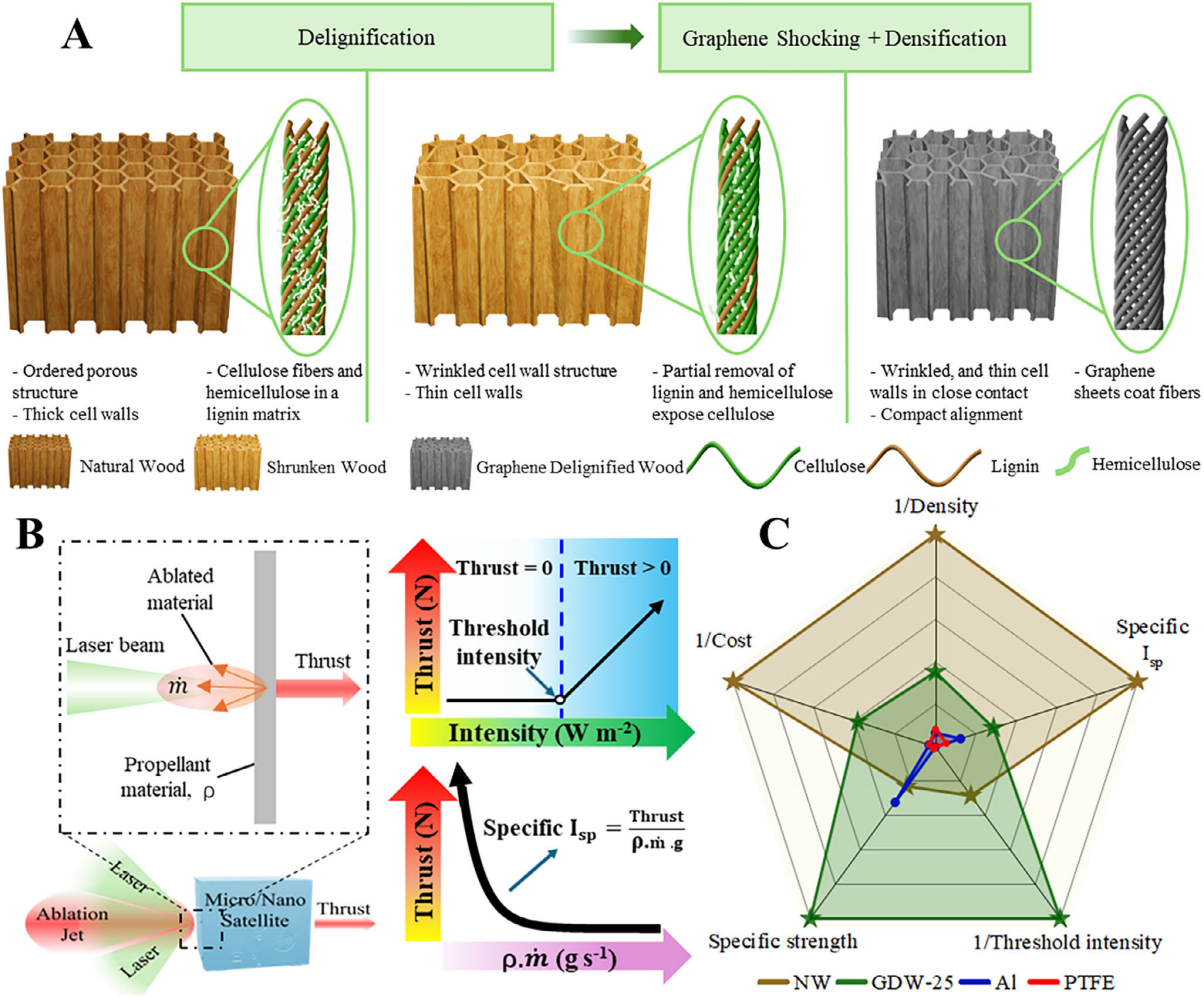
Preparation of GDW, LAP application, and comparison with common materials. (A) Illustration of the preparation of GDW composites. (B) Application as a propellant material for LAP in micro/nano satellites. (C) Radar plot comparing the performance of NW and GDW‐25 with Al and Polytetrafluoroethylene (PTFE), in which the results are normalized by the maximum value of each characteristic.

Graphene is produced through microwave‐assisted expansion of intercalated graphite (IG) to form expanded graphite (EG), followed by high‐pressure homogenizer (HPH) exfoliation. NW (Figure 1A) is delignified using a 2.5 m NaOH 0.4 Na_2_SO_3_ solution over a varying range of durations, denoted by “x”. In the NaOH / Na_2_SO_3_ solution, the lignin‐carbohydrate complex (LCC) undergoes cleavage through two mechanisms [54]. OH^−1^ cleaves ester linkages between hemicellulose and lignin, and SO_3_^−2^ undergoes nucleophilic attack at the phenolic hydroxyl groups on lignin's aromatic rings, cleaving methyl‐aryl ether bonds and generating water‐soluble lignosulfonate. SO_3_^−2^ can also induce cleavage of ß─O─4 bonds, further facilitating lignin sulfonation. Hemicellulose is partially removed due to the hydrolysis of glycosidic bonds within polysaccharide chains. This process partially removes lignin and hemicellulose while maintaining the hierarchically anisotropic cellulose framework structure. Moreover, the specific concentration of 2.5 m NaOH/0.4 m Na_2_SO_3_ has been shown to produce the highest crystallinity index of cellulose, leading to the best mechanical performance [55]. The partial removal of this hydrophobic component results in softening and mild expansion in the wood sample size due to the absorption of water by the remaining cell walls with a higher fraction of hydrophilic cellulose [56]. Treated wood is then washed with deionized water and let dry, forming an array of shrunken wood (Figure S1). Shrunken wood is then “shocked” with the graphene dispersion as per the work by Chen et al. [56]. The shocking process entails immersing the shrunken wood for 3 min in a graphene dispersion, which partially re‐swells the cell walls. The resulting wood is highly foldable in directions both parallel and perpendicular to fiber orientation, without breaking (Videos SV1 and SV2). Shocked wood is then densified by applying pressure during oven‐drying to form GDW. Rapid moisture evaporation and compression during densification induce shrinkage of the wood sample. This densification method promotes additional formations of hydrogen bonding among the cellulose nanofibrils, whose increased specific area and exposed hydroxyl groups from delignification [54, 55]. Moreover, the exposed cellulose nanofibrils interact with graphene through cation–π interactions formed between Na^+^ in the SDC dispersing medium in the graphene dispersion and π‐electron clouds on the surface of graphene [57]. To assess the effect of graphene on LAP performance, shrunken wood was also shocked with de‐ionized (DI) water to form delignified wood (DW). The samples are denoted by DW‐x or GDW‐x, where x represents the number of hours of delignification treatment. These samples can be utilized as LAP propellants for micro/nano satellites, where the threshold intensity, the minimum intensity required to initiate ablation, and density‐specific *I*_sp_ are key parameters (Figure 1B). Experimental results show that NW displayed an ablation threshold intensity of 1.9 MW m^−2^ and an *I*_sp_ of 907.74 s with a density of 0.18 g cm^−3^, resulting in a specific *I*_sp_ of 5043.0 ± 188 s g^−1^ cm^3^. However, NW yielded a relatively low tensile strength of 22.79 MPa (Figure S2). Delignification and graphene enhancement retain the LAP performance while improving the tensile strength. GDW‐25 achieved an *I*_sp_ of 800.49 ± 28.51 s with a density of 0.51 g cm^−3^, leading to a specific *I*_sp_ of 1569.60 ± 57.40 s g^−1^ cm^3^. It also displayed an ultra‐low ablation threshold intensity of 0.54 MW m^−2^, the lowest seen in the literature. GDW‐25 achieved a tensile strength of 273.1 MPa and a specific strength of 533.33 MPa g^−1^ cm^3^, exceeding that of steel (∼89.4 MPa cm^3^ g^−1^) [58] and Aluminum (84–172 MPa cm^3^ g^−1^) [59] alloys widely used in aerospace structures. Wood and graphene–wood composites displayed favorable LAP properties, strength‐to‐weight ratios, and cost (Table S1) compared with widely used aerospace materials (Figure 1C; Table S2). Higher performance parameters could be achieved by tuning the cellular structure and optimizing laser–matter interactions, opening up new avenues for future research and application in laser propulsion.

**FIGURE 2:**
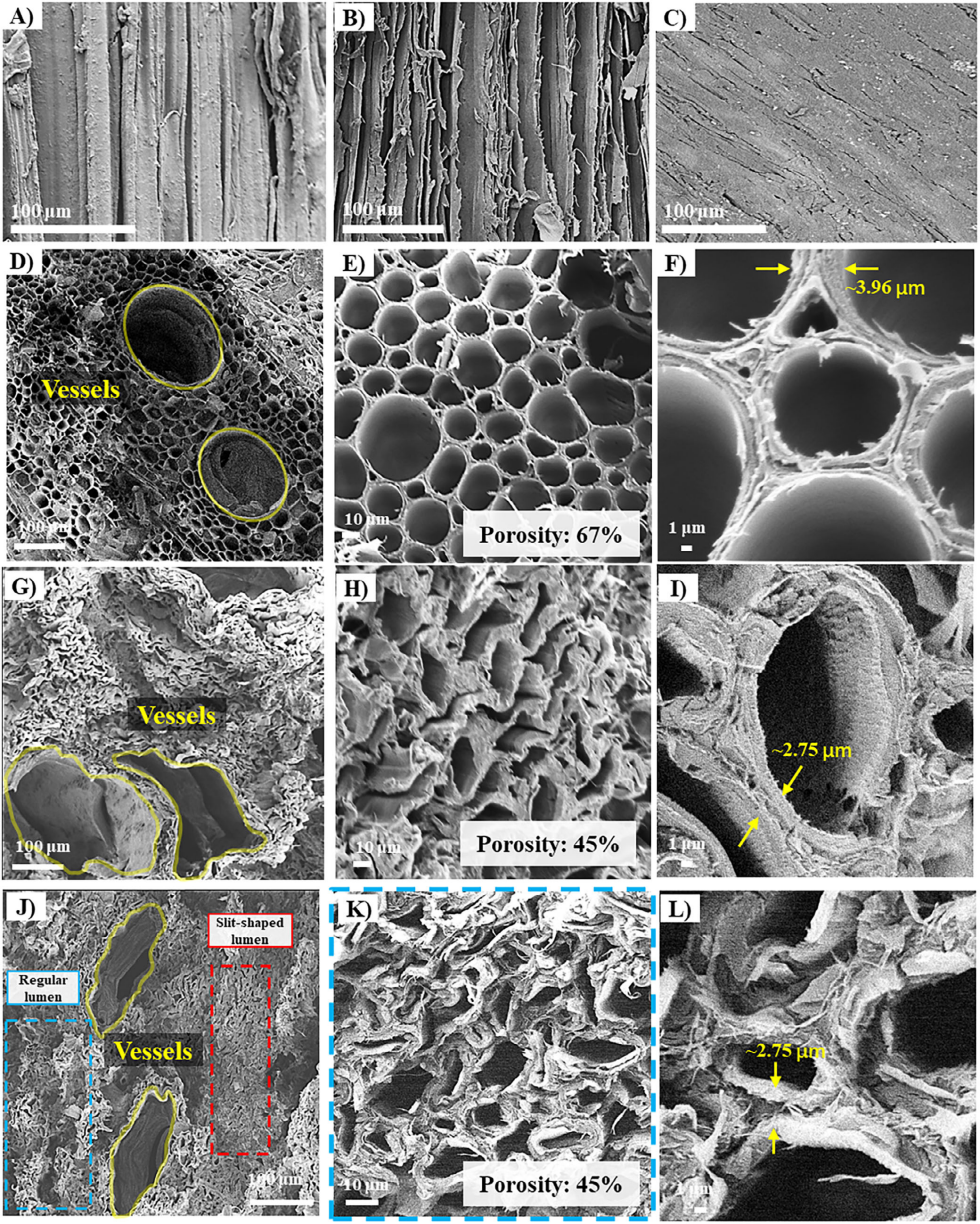
SEM images and porosity and cell wall thickness analysis of NW, DW, and GDW. SEMs of the tangential view of (A) NW, (B) DW‐25, and (C) GDW‐25 and the cross‐sectional view showing vessels and lumina, porosity, and average cell wall thickness of (D–F) NW, (G–I) DW‐25, and (J–L) GDW‐25.

Scanning electron microscopy (SEM) analysis is performed to assess the properties of microstructures of the samples, including cell wall thickness and porosity, which have been shown to affect light scattering in wood [60] and its mechanical performance [56]. SEM analysis of cell wall shape and connectivity in the cell's corners has also been shown to affect the flexibility of wood composites [56]. SEM images of the tangential view of the surface of NW (Figure 2A) and DW‐25 (Figure 2B) demonstrate that the removal of lignin from the matrix leads to a denser and more compact alignment of wood fibers. The SEM image of the tangential view of GDW‐25 (Figure 2C) reveals a uniform coating of graphene on the surface, while the cross‐sectional view of NW (Figure 2D) reveals an ordered porous structure of vessels and lumina (vertical cellular voids) with a porosity of 67% (Figure 2E) and a cell wall thickness of 3.96 µm (Figure 2F).

**FIGURE 3:**
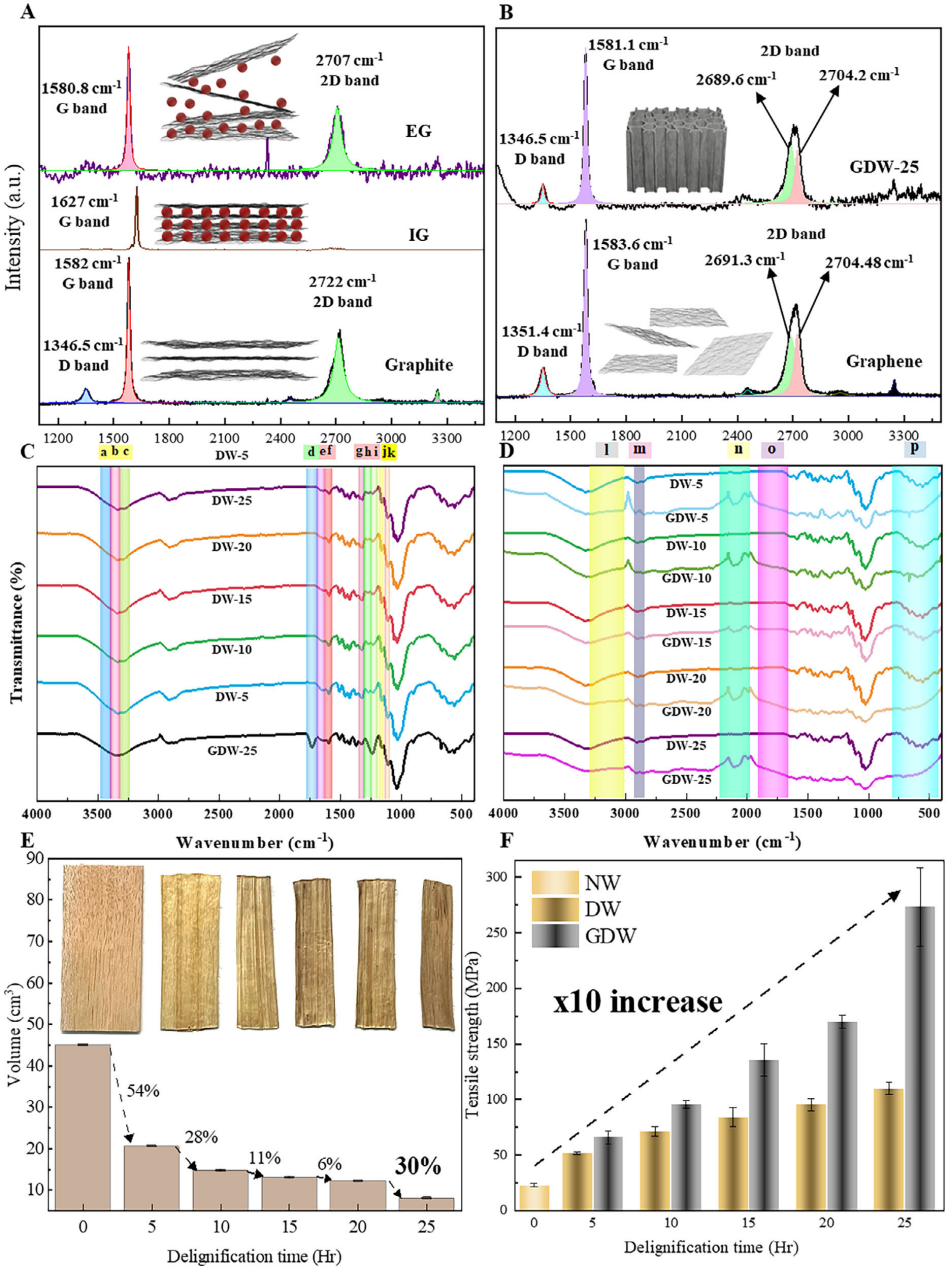
Characterization of NW, DW‐x, and GDW‐x. (A) Raman spectra of EG, IG, and graphite, where red dots represent HClO_4_ ions and grey mesh represents carbon sheets. (B) Raman spectra of graphene and GDW‐25. (C) FTIR spectra of NW and DW‐x. (D) FTIR spectra of DW‐x and GDW‐x. (E) Volume reduction with delignification time. (F) Tensile strengths parallel to the fiber direction of NW, DW‐x, and GDW‐x composites.

On the contrary, DW‐25 and GDW‐25 (Figure 2G,J) reveal a “wrinkled” cell wall structure with open vessels and partially open lumina. The shocking process prevents the separation of cell walls, allowing sufficient hydrogen bonding among cell walls to resist delamination during folding [56]. As a result, the wrinkled cell wall structure can support high strain, allowing the material to be easily folded, both in the direction parallel and perpendicular to wood fibers, into the desired shape without breaking (Figure S3). DW‐25 and GDW‐25 display a lower overall porosity of 45% (Figure 2H,K) and a lower cell wall thickness of 2.75 µm (Figure 2I,L) than NW. The decrease in porosity despite a reduction in cell wall thickness may be attributed to the presence of partially closed lumina within the structure, forming areas of “slit‐shaped” lumina with a porosity of 19% (Figure S4) in comparison with regular lumina. Raman analysis of graphite, IG, and EG (Figure 3A) confirms the successful intercalation and expansion of graphite (Discussion S1), while the Raman analysis of graphene and GDW‐25 (Figure 3B) shows an *I*_2D_/*I*_G_ ratio of ∼0.53, confirming the successful formation of multilayered graphene sheets from EG and their deposition on wood's surface. FTIR analysis of NW and DW‐x (Figure 3C) confirms that the removal of lignin and hemicellulose by delignification increases with delignification time (*x*, hrs), which explains the shrinkage in samples with delignification time (Figure 3E; Discussion S2). FTIR analysis of DW‐x and GDW‐x shows a band broadening and shift corresponding to graphene–wood interaction, which also increases with delignification time (Figure 3D), which explains the increase in tensile strength with graphene enhancement (Discussion S3). Mechanical tensile tests reveal that the delignification and densification processes resulted in an increase in tensile strength with delignification time from 22.79 MPa of NW to 109.73 MPa of DW‐25, and to 273.13 MPa of GDW‐25, where GDW exhibits over an order of magnitude higher tensile strength than natural wood (Figure 3F). The improved mechanical properties of DW are due to its modified microstructure, which features highly packed intertwined cell walls at the microscale. The hot‐pressing densification process densified the porous wood cells and increased the internal friction between them, resulting in a tightly intertwined cell wall with additional inter/intramolecular hydrogen bonds between neighboring nano‐fibrils, which collectively enhance the mechanical properties of the wood [61]. The improved mechanical properties of GDW are due to graphene bonding with the exposed cellulose fibrils, enhancing stress distribution.

**FIGURE 4:**
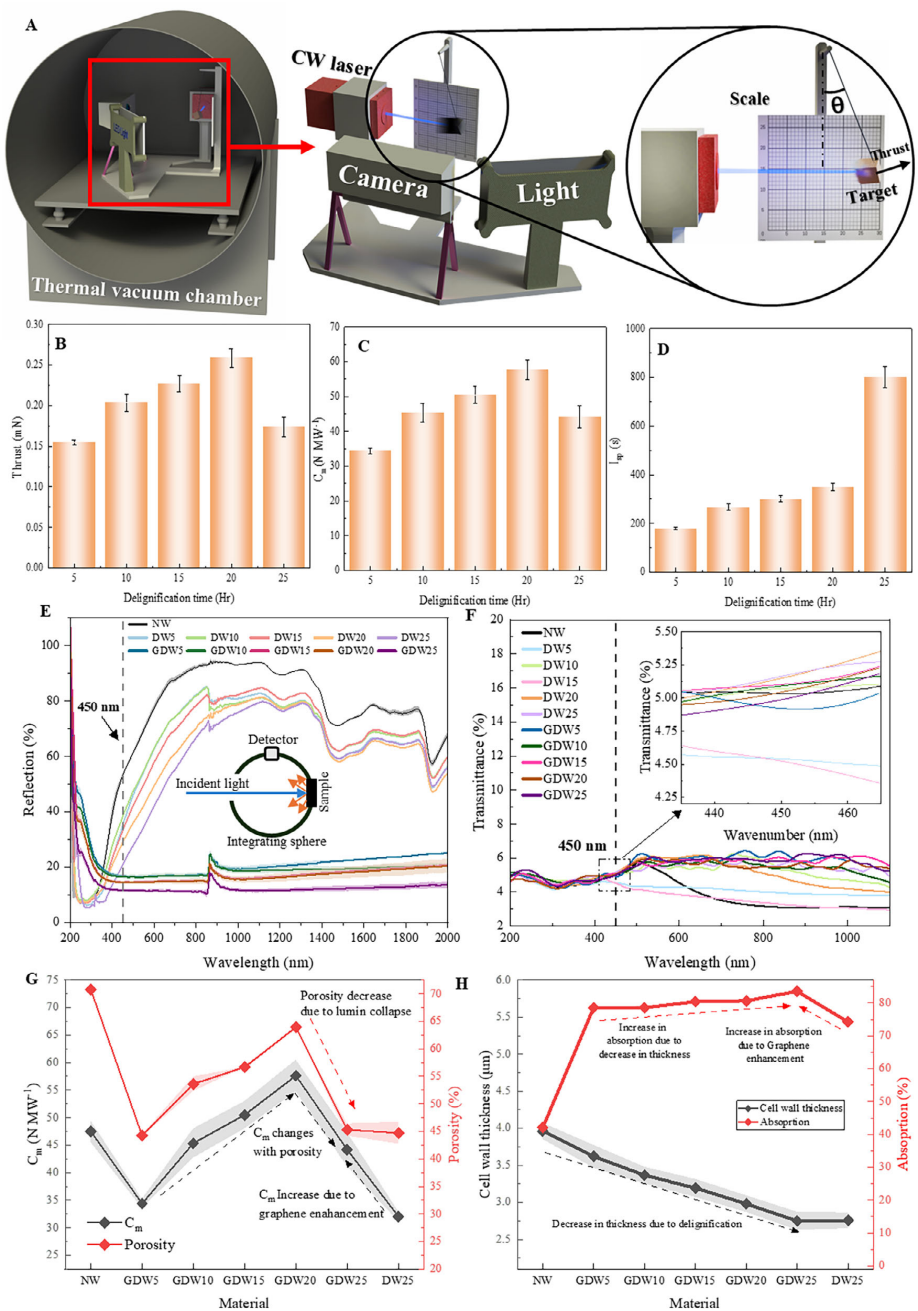
LAP experimental setup, results, and characterization for GDW‐x. (A) Experimental setup inside the thermal vacuum chamber (TVAC) for thrust force measurement. Results of (B) thrust, (C) *C*_m_, and (D) *I*_sp_ of the samples. UV–vis spectra of the (E) reflection and (F) transmission of the samples, showing the effect of delignification time and graphene enhancement. (G) Comparison showing a positive correlation between the porosity and *C*_m_. (H) Comparison showing a positive correlation between the cell wall thickness and *I*_sp_.

The hanging pendulum method [62] was employed to compare and quantify the LAP performance parameters of GDW‐x targets in vacuum (Figure 4A) by utilizing a CW laser at an intensity of 5.01 MW m^−2^ (Video SV3). The pendulum motion of the samples was tracked and analyzed for thrust performance (Figures S5 and S6). SEM analysis of the average porosity and cell wall thickness of GDW‐x targets is shown in Figure S7. As delignification time increased from 5 to 20 h, a steady increase in thrust was observed (Figure 4B), peaking at 0.259 ± 0.004 mN for GDW‐20. This was accompanied by a maximum *C*_m_ of 57.60 ± 0.97 N MW^−1^ (Figure 4C), indicating superior impulse transfer efficiency. Beyond 20 h, however, performance began to degrade. For GDW‐25, thrust dropped to 0.172 ± 0.004 mN and *C*_m_ dropped to 44.23 ± 0.97 N MW^−1^. The change in performance due to delignification is the result of changes in the microstructure, porosity, and cell wall thickness, and consequent changes in the total reflection (Figure 4E) and transmission (Figure 4F) spectra of the samples, from which the absorption at λ = 455 nm is measured. *C*_m_ is governed by the directed ejection of ablated mass and, consequently, efficient conversion of laser energy to momentum. The trend of an increase in *C*_m_ up to GDW‐20 closely mirrors the progression in porosity (Figure 4G), which also reaches a maximum at GDW‐20 and drops at GDW‐25. The increase in porosity up to 20 h is due to partial removal of lignin and hemicellulose. The decrease in porosity after 20 h may be attributed to the presence of compressed “slit‐shaped” partially closed lumina (Figure S4), caused by 30% reduction in volume (Figure 3B). This trend suggests that moderate delignification enhances the material's internal porosity and vapor channeling, enabling improved ablation jet confinement and more efficient laser energy conversion into momentum. *I*_sp_ is governed by efficient laser energy absorption and conversion to heat and, consequently, reduced mass loss rates. *I*_sp_ value increases consistently with delignification time and reaches a maximum of 800.49 ± 28.51 s at GDW‐25 compared to 350.83 ± 22.79 s at GDW‐20 (Figure 4D). GDW‐25 also displays a lower mass consumption rate than GDW‐20 (Figure S8). The trend in ablation efficiency (*η*) is similar, with a maximum of 17% at GDW‐25 compared to 10% at GDW‐20 (Figure S9). This trend is consistent with the cell wall thickness and absorption coefficients of the samples (Figure 4H).

The increased absorption with delignification time is the result of thinner cell walls due to lignin and hemicellulose partial removal during delignification. The reduction in cell wall thickness reduces the refractive index (RI) inhomogeneities across the wood, and consequently, the anisotropic light scattering according to the Maxwell–Garnett approximation [63, 64, 65], trapping light within the inner structure of the wood and, hence, increasing its absorption. The positive correlation between cell wall thickness and total reflectance was also observed in Zhao et al. [66]. Thinner cell walls increase light confinement, transferring more energy to ablated materials, resulting in more energetic particles ejected at greater exhaust velocities (**v**_ex_). The increased light confinement also explains the reduction in crater depth after laser irradiation (Figure S10). Up to a delignification time of 20 h, the increase in *I*_sp_ is also consistent with the increase in porosity, which favors light trapping due to the lower volume‐filling of the solid cell walls that are defined as light scatterers [63, 67]. Beyond 20 h, the increase in *I*_sp_ at GDW‐25, despite the drop in porosity, indicates a shift in the heat conversion. The lower porosity indicates a large number of solid–solid interfaces and a higher volume filling fraction, while thinner cell walls lower the refractive index mismatch and hence light scattering between the void in the lumen and the cell walls. According to Snell's law [68, 69], this results in higher light attenuation [70], i.e., transfer of light energy to heat energy, as explained in Li et al. [65]. The positive correlation between solid–solid interfaces and light attenuation in DW composites was also observed by Anish et al. [71]. and explained by the Beer‐Lambert law (Figure S11) [72]. These results emphasize two key principles of cell wall engineering for LAP of wood composites. First, material ablation must be enhanced through absorption to enhance ablation, reduce mass consumption rate, and increase *I*_sp_. Absorption is enhanced by the addition of graphene and by thinner cell walls. Second, mass ejection must be axially directed to maximize momentum transfer and hence thrust and *C*_m_. Enhanced axial mass ejection is achieved through increased porosity. Tuning of the porosity and cell wall thickness through delignification time serves as a cell wall engineering strategy to tune between mechanical recoil‐dominated propulsion (optimized at GDW–20) and high‐*I*_sp_ propulsion (optimized at GDW‐25). These insights confirm that delignification time serves as a controllable parameter to optimize LAP performance for targeted mission profiles. Such understanding holds significant implications for the rational design and engineering of advanced sustainable materials for diverse applications in photonic propulsion and beyond [65].

**FIGURE 5:**
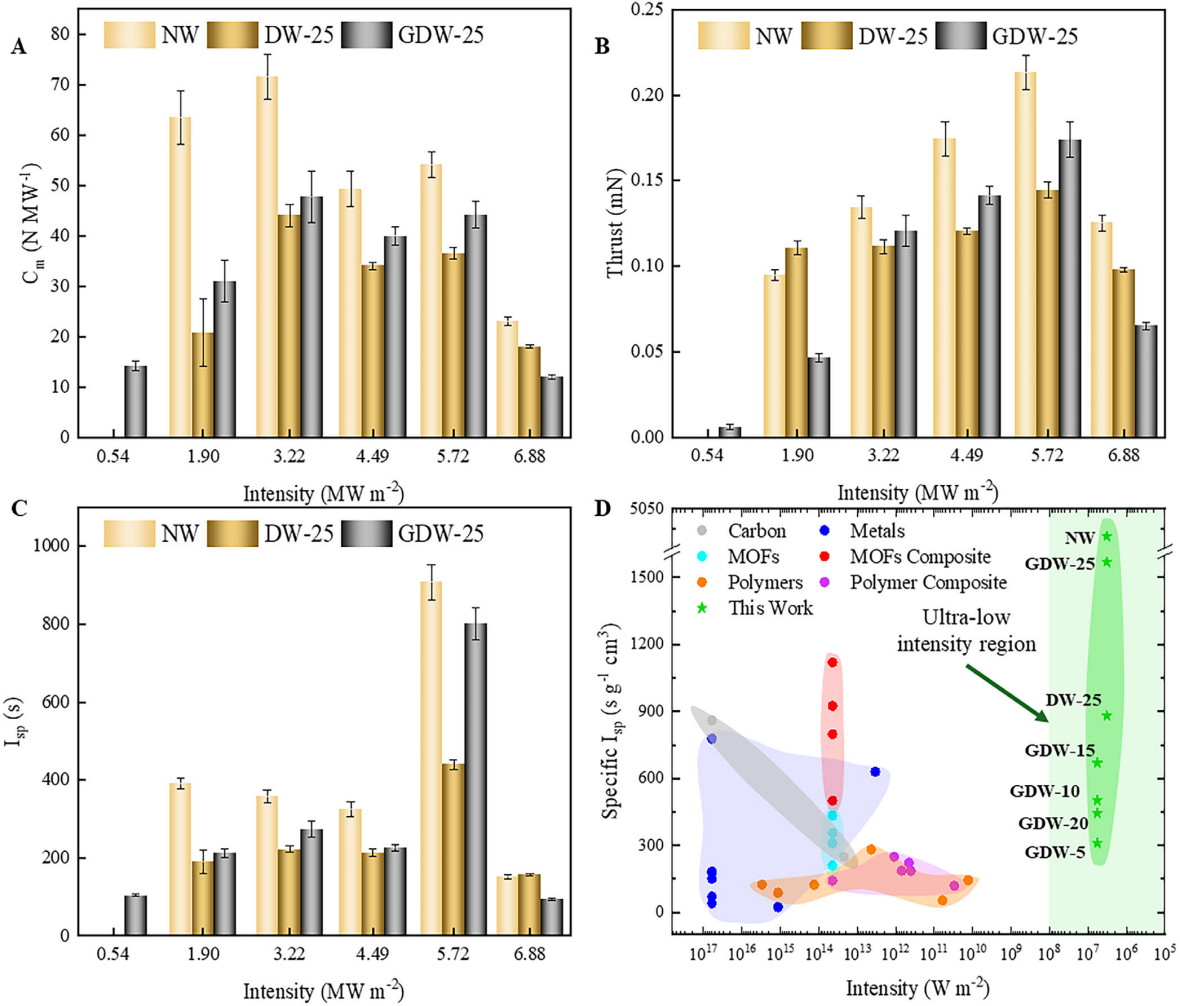
NW, DW‐25, and GDW‐25 LAP results at different intensities and comparison with previous works. Results of (A) *C*_m_, (B) Thrust, and (C) *I*_sp_ of the samples at different laser intensities. (D) Comparison of specific *I*_sp_ and intensity with other material types.

LAP results for NW, DW‐25, and GDW‐25 at laser intensities varying from 0.54 to 6.88 MW m^−2^ are analyzed to understand the effect of delignification and graphene enhancement on LAP performance (Video SV4). *C*_m_ initially increases with intensity, until it reaches an optimum value (*C*_m, opt_) after which it starts decreasing (Figure 5A). At an intensity of 2.31 MW m^−2^, DW‐25 demonstrated the smallest *C*_m,opt_ of 44.10 ± 1.72 N MW^−1^, followed by 47.80 ± 1.08 N MW^−1^ of GDW‐25, and a maximum *C*_m,opt_ of 72 ± 1.20 N MW^−1^ for NW. In Phipps et al. [73]. It was shown that all target materials follow a similar *C*_m_ versus intensity trend for metals and polymers under pulsed LAP. For NW, DW‐25, and GDW‐25, the trends for thrust (Figure 5B) and *I*_sp_ (Figure 5C) are similar, but peak at different intensities. For instance, at 5.72 MW m^−2^, the maximum thrust of 0.1487 ± 0.006, 0.172 ± 0.004, and 0.213 ± 0.004 mN, and the maximum *I*_sp_ of 439.92 ± 18.74, 800.49 ± 28.51, and 907.74 ± 30.53 s are obtained for DW‐25, GDW‐25, and NW, respectively. Similarly, peaked at an intensity of 5.72 MW m^−2^, reaching 8%, 17%, and 25% for DW‐25, GDW‐25, and NW, respectively (Figure S12). Comparisons are made between NW and GDW‐25 composites and other LAP propellant materials reported in the literature, which clearly demonstrate that the developed materials exceed those materials previously documented, in terms of specific *I*_sp_ and ablation intensity (Figure 5D; Table S3). These results unveil, for the first time, an entirely new regime of CW LAP; where NW, DW‐25, and GDW‐x composites achieve absolute *I*_sp_ values at ultra‐low intensity regions (Figure S13), which has never been achieved with either pulsed or CW LAP before. Additionally, GDW‐25 produces thrust levels ∼2 and 3–4 orders of magnitude larger than graphene sails [46, 74, 75] and graphene aerogels [47, 48, 50], respectively, with comparable *C*_m_ values (Table S4). The primary reason for this difference lies in the propulsion mechanism. Whereas previously reported graphene propulsion systems rely primarily on laser‐induced thermal gradients in rarefied gas environments, the GDW material operates through laser ablation propulsion, where thrust is generated by direct momentum recoil from ejected material. This ablation‐driven mechanism enables substantially greater momentum transfer.

**FIGURE 6:**
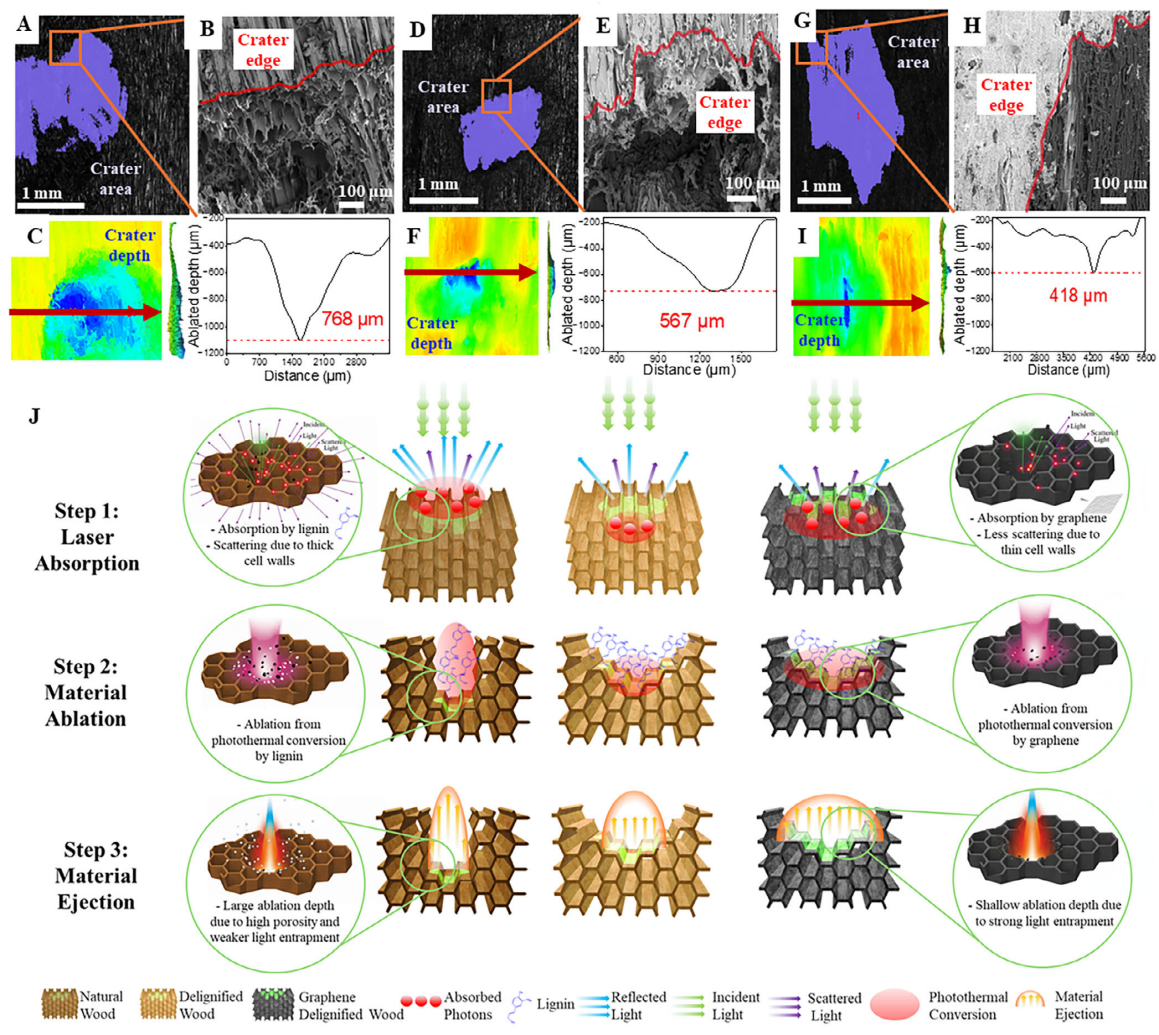
Comparison between NW, DW‐25, and GDW‐25's surface morphology after laser irradiation and LAP mechanism. Profilometer images of ablated crater areas, SEM of ablated crater edges, and profilometer images of the crater depth of (A–C) NW, (D–F) DW‐25, (G–I) GDW‐25. (J) Illustration of light–material interaction in LAP at three process steps for NW, DW‐25, and GDW‐25.

At peak *I*_sp_ intensity, we investigated the targets’ surface morphology following 3 s of laser interaction. Laser profilometer imaging reveals a decrease in crater area, the total area of the ablation crater, from 1.11 mm^2^ for NW (Figure 6A) to 0.72 mm^2^ for DW‐25 (Figure 6D), but an increase to 2.49 mm^2^ for GDW‐25 (Figure 6G). Profilometer analysis of the ablation depth shows a relatively large depth of 0.77 mm for NW (Figure 6C), compared to 0.57 mm for DW‐25 (Figure 6F) and 0.42 mm for GDW‐25 (Figure 6I). UV–vis spectra reveal a 32.32% reduction in total reflection from NW to DW‐25, consistent with the 30.39% reduction in cell wall thickness (Figure S14). This indicates an entrapment of light within the inner structure and explains the reduced crater depth and area of DW‐25 compared to NW according to the Beer‐Lambert law [70]. DW‐25 has a lower thrust and *C*_m_ than NW despite a greater mass loss rate of 33.5 µg s^−1^, indicating hindered axial confinement due to lower porosity (Figure S15). DW‐25 also has a relatively rough crater edge compared to NW (Figure 6E,H), indicating bigger ejected particles. A similar correlation between crater edge and ejected particle size was made by Rao et al. [20]. This may be attributed to the greater amount of lignin within the structure of NW, which is the main component of wood responsible for light attenuation within cell walls and photothermal conversion [69, 76]. Lignin's low thermal conductivity utilizes heat energy for material ablation and ejection. The enhanced *I*_sp_ performance of GDW‐25 compared to DW‐25 may be attributed to the restored thermal conversion of the graphene layer and its superior light absorption via π‐band optical transitions [52, 64, 66, 75]. Graphene moves heat exceptionally well due to its high thermal conductivity, so the deposited energy quickly becomes and spreads as heat. GDW‐25 exhibits the greatest thermal conductivity at 0.12 W m^−1^K^−1^ compared to 0.03 W m^−1^K^−1^ and 0.07 W m^−1^K^−1^ for NW and DW‐25, respectively. The graphene layer efficiently absorbs and deposits light and thermal energy onto the wood layer, while the wood structure entraps light, enhancing light–matter interactions. As a result, GDW‐25 has the lowest mass loss rate (22.2 µg s^−1^) (Figure S15), with the shallowest crater depth and the largest crater area. This pattern points to a lateral spread of ablation facilitated by graphene‐enhanced thermal diffusion, explaining the relatively smoother crater edge (Figure 6H). The inferred light–matter interaction and its corresponding LAP mechanism is illustrated in Figure 6J. Furthermore, the consistently low reflection of GDW‐25 across the UV‐VIS range (Figure 4E) suggests consistent LAP performance at higher laser wavelengths, favored for space environment applications.

To determine whether bulk sample thickness, which differs slightly from specimen to specimen, has an effect on propulsion parameters, several specimens of NW, DW‐25, and GDW‐25 with varying sample thickness were tested under identical conditions: intensity of 5.72 MW m^−2^, pressure of 2.5 × 10^−4^ Torr. The coefficient of variation of sample mass was kept below 2.5%. No direct correlation between sample thickness and propulsion parameters was observed, indicating that bulk sample thickness does not affect resultant performance (Figure S16). Previous studies on the long‐term exposure of wood to space environment conditions, such as outgassing [38, 77] and radiation [78, 79, 80] demonstrate promising stability in space. The mechanical and propulsive performance of GDW under long‐term exposure to such conditions should be investigated in future studies.

## 3 Conclusion

This paper presented a CW LAP concept by utilizing NW and GDW composites as next‐generation propellants for space applications. The synergistic combination of low thermal conductivity of wood with high energy absorption of graphene yielded high‐performance GDW‐25 with an ultra‐low ablation threshold intensity at 0.54 MW m^−2^ and a specific *I*_sp_ at 1569.60 ± 57.40 s g^−1^ cm^3^, surpassing reported values in the literature. These results imply that, for a given volume, the developed GDW propellant delivers the same total impulse with much less mass. This high energy density property is especially appealing for weight‐constrained micro/nano satellite missions. It was also demonstrated for the first time the largest attainable absolute *I*_sp_ value of 800.49 ± 28.51 s for CW laser irradiation mode, where even achieving an *I*_sp_ above 100 s has been proved a significant challenge. By fine‐tuning the cellular structure of the wood and optimizing light–material interactions, higher LAP performance can be expected, thereby opening a new avenue for future research in laser propulsion applications. These findings summarize the criticalities of GDW and wood‐based composites as potential next‐generation space materials, enabling low‐mass, ultra‐low power, and sustainable space mission designs that are previously unachievable with micro/nano satellites.

## Conflicts of Interest

The authors declare no conflicts of interest.

## Supporting information




**Supporting File 1**: advs75463‐sup‐0001‐SuppMat.docx.

**Supporting File 2**: advs75463‐sup‐0002‐Video S1.mp4.

**Supporting File 3**: advs75463‐sup‐0003‐Video S2.mp4.

**Supporting File 4**: advs75463‐sup‐0004‐Video S3.mp4.

**Supporting File 5**: advs75463‐sup‐0005‐Video S4.mp4.

## Data Availability

The data that support the findings of this study are available from the corresponding author upon reasonable request.
