# Supplementary material for: Ultra‐Low Intensity Continuous Wave Laser Ablation Propulsion With Graphene‐Engineered Wood
Source: Adv Sci (Weinh). 2026 May 16;13(42):e75463. doi: 10.1002/advs.75463 (PMC13335535; doi:10.1002/advs.75463)
Supplement: Supplementary file 1 — Supporting File 1: advs75463‐sup‐0001‐SuppMat.docx. [file ADVS-13-e75463-s005.docx]

**SUPPLEMENTARY INFORMATION**

Ultra-Low Intensity Continuous Wave Laser Ablation Propulsion with Graphene-Engineered Wood

*Afnan S. M. Elmubasher^a,b^, Rami Elkaffas^a^, Mohamed Hamid Salim^c,d^, Basel Altawil^a^, Chanaka Sandaruwan^a,e^, Shanavas Shajahan^e^, Ahsan Baidar Bakht^f^, Irfan Hussain^f^, Blaise L. Tardy^c,d^, Sean S. M. Swei^a,b^, Yarjan Abdul Samad^a,g,h^*

1. *Department of Aerospace Engineering, Khalifa University, Abu Dhabi, United Arab Emirates.*
2. *Khalifa University Space Technology and Innovation Lab, Khalifa University, Abu Dhabi, United Arab Emirates.*
3. *Department of Chemical Engineering, Abu Dhabi, United Arab Emirates, Khalifa University, Abu Dhabi, United Arab Emirates.*
4. *Food Security and Technology Center, Khalifa University, Abu Dhabi, United Arab Emirates.*
5. *Research and Innovation Centre on 2D Materials, Khalifa University, Abu Dhabi, United Arab Emirates.*
6. *Department of Mechanical and Nuclear Engineering, Khalifa University, Abu Dhabi, United Arab Emirates.*
7. *Department of Electrical Engineering, University of Cambridge, Cambridge, United Kingdom.*
8. *Advanced Research and Innovation Center (ARIC), Khalifa University, Abu Dhabi, United Arab Emirates.*

* To whom correspondence should be addressed

E-mail: [yarjan.abdulsamad@ku.ac.ae](mailto:yarjan.abdulsamad@ku.ac.ae), [yy418@cam.ac.uk](mailto:yy418@cam.ac.uk)

Contents

[Supplementary Discussions 3](#_Toc224647881)

[*Supplementary Discussion 1: Raman analysis of graphite, IG, EG, graphene, and GDW-25 3*](#_Toc224647882)

[*Supplementary Discussion 2: FTIR analysis of NW, DW-x and GDW-x composites 4*](#_Toc224647883)

[*Supplementary Discussion 3: Mechanical properties 5*](#_Toc224647884)

[Characterization Methods 5](#_Toc224647885)

[Materials and Experimental Setup 7](#_Toc224647886)

[Chemicals: 7](#_Toc224647887)

[Synthesis Procedures: 7](#_Toc224647888)

[Propulsion Tests: 7](#_Toc224647889)

[Calculations 8](#_Toc224647890)

[Sample density: 8](#_Toc224647891)

[Propulsion Parameters: 9](#_Toc224647892)

[Attenuation coefficient: 10](#_Toc224647893)

[Uncertainty and error propagation: 10](#_Toc224647894)

[Cost of materials: 15](#_Toc224647895)

[Supplementary Figures 17](#_Toc224647896)

[Figure S1. Digital images of the DW samples before and after drying. A) DW-x. B) Shrunken wood. 17](#_Toc224647897)

[Figure S2. Tensile test results of NW, DW-x, and GDW-x composites. The shaded region represents the standard deviation. 17](#_Toc224647898)

[Figure S3. Digital images of MW samples post-shocking with DI water and graphene. Samples folded by ~180° parallel to fiber direction, by ~180° perpendicular to fiber direction, into a spiral parallel to fiber direction, and into a random shape parallel to fiber direction of A-D) DW and E-H) GDW. 18](#_Toc224647899)

[Figure S4. SEM images of GDW showing slit-shaped lumina region. A) the graphene layer and “slit-shaped” lumina region with a porosity of 19% and B) cell wall thickness of ~2.75 μm. 18](#_Toc224647900)

[Figure S5. Digital images of the tracked sample movement in pendulum motion with time. A) NW, B) DW, and C) GDW-25 targets. 19](#_Toc224647901)

[Figure S6. SeqTrack workflow. The tracker uses a template image and processes each frame as a search image. Regions are converted into embeddings, and the encoder-decoder transformer predicts bounding boxes to generate the object’s continuous trajectory. 20](#_Toc224647902)

[Figure S7. SEMs showing cross sections with vessels (highlighted in yellow), porosities of lumina, and cell wall thicknesses of different delignification times. A-C) GDW5, D-F) GDW10, G-I) GDW15, and J-L) GDW20. 21](#_Toc224647903)

[Figure S8. Mass loss rate vs delignification time for GDW-x composites. 22](#_Toc224647904)

[Figure S9. Ablation efficiency vs delignification time for GDW-x composites. 22](#_Toc224647905)

[Figure S10. Crater area, edge, and depth analyses of GDW-x composites. Profilometer images of ablated crater areas, SEM of ablated crater edges, and profilometer images of the crater depth of A-C) GDW-5, D-F) GDW-10, G-I) GDW-15, and J-L) GDW-20. 23](#_Toc224647906)

[Figure S11. Attenuation coefficient and porosity for GDW-x composites. 24](#_Toc224647907)

[Figure S12. Ablation efficiency vs laser intensity for NW, DW-25, and GDW-25. 24](#_Toc224647908)

[Figure S13. Comparison of I_sp_ and intensity with other material types. 25](#_Toc224647909)

[Figure S14. Comparison between microstructure and optical parameters of NW, DW-25, and GDW-25. 26](#_Toc224647910)

[Figure S15. Mass consumption rate vs laser intensity for NW, DW-25, and GDW-25. 26](#_Toc224647911)

[Figure S16. Propulsion parameters vs sample thickness. A) NW, B) DW-25, and C) GDW-25. 26](#_Toc224647912)

[Figure S17. FTIR spectra of the surfactant used, dried graphene ink powder, delignified wood (DW-25), and graphene ink-coated delignified wood (GDW-25). 27](#_Toc224647913)

[Supplementary Tables 27](#_Toc224647914)

[Supplementary Table 1: Cost of materials of NW, DW-25, GDW-25, Al 7075-T6, and PTFE. 27](#_Toc224647915)

[Supplementary Table 2: The performance comparison of structural materials. 28](#_Toc224647916)

[Supplementary Table 3: The performance comparison of different propellants. 28](#_Toc224647917)

[Supplementary Table 4: Comparison between GDW and previous graphene materials' performance in light propulsion. 30](#_Toc224647918)

[Supplementary Table 5: Overall functional group assignments regarding cellulose, hemicellulose, and lignin components of wood. 30](#_Toc224647919)

[Supplementary References 31](#_Toc224647920)

Supplementary Information

# **Supplementary Discussions**

## **Supplementary Discussion 1: Raman analysis of graphite, IG, EG, graphene, and GDW-25**

The prepared materials were further studied with the Raman analysis, where the Raman spectra of Graphite, IG, EG, Graphene ink, and GDW are shown in Fig. 2a. The typical G peaks responsible for the in-plane vibrations of carbon atoms and 2D peaks responsible for the lattice double resonance process in the graphite structure are located at 1582 and 2722 cm^-1^.^1^ The intercalation of graphite with perchloric acid in between the carbon layers of pristine graphite is confirmed with the peak shifting of the G band towards a higher wavenumber at 1627 cm^-1^ in the Raman spectra of IG. The peak shift of around 45 cm^-1^ confirms the success of intercalation.^2^ The Raman spectra of EG show a right shift in the G band and a left shift in the 2D band at 1580.8 and 2707 cm-1, respectively, when compared with the pristine graphite.^3^ The observed shift is due to the van der Waals force reduction between the carbon layers. The 2D band in the Raman spectra of graphene ink clearly shows the peak shift towards lower wave number at around 2691.3 cm^-1^, showing the reduction in the number of carbon layers, and the G band also showed a distinct higher wavenumber shift at 1583.6 cm-1, confirming the successful exfoliation of graphite into graphene sheets. The ratio of 2D and G peak intensities gives information on the number of layers in graphene, whereas I_2D_/I_G_ of graphene ink is found to be ~0.53, confirming the formation of multilayered graphene sheets.^4^ Further, the Raman spectra of GDW show similar peaks to those of the graphene ink, which shows that the properties of graphene are not affected even after coating it on the surface of delignified wood.

## **Supplementary Discussion 2: FTIR analysis of NW, DW-x and GDW-x composites**

The FTIR analysis confirms that the delignification treatment (DW5–DW25) primarily removed lignin and hemicellulose while leaving cellulose intact. The characteristic cellulose absorption bands at 3400, 3330, and 3270 cm⁻¹ (assigned to –OH stretching, **Figure 3**C.a,b, and c, respectively)^5,6^ as well as 1150 and 1103 cm⁻¹ (C–O–C and C–O stretching vibrations,^7,8^ **Figure 3**C.j,k respectively) remained unchanged throughout all samples, indicating that the cellulose backbone was preserved during the process. In contrast, the hemicellulose-associated carbonyl band stretching (**Figure 3**C.d) at 1730 cm⁻¹^9,10^ disappeared completely after only 5 hours of delignification time (DW-5). It was absent in all subsequent samples (DW10–DW25), demonstrating effective and rapid removal of hemicellulose. For lignin, while the aromatic ring vibrations (**Figure 3**C.f) at 1590 cm⁻¹ and the C=C stretching (**Figure 3**C.e) at 1654 cm⁻¹ remained constant across all delignification times, the typical C–O stretching band (**Figure 3**C.h+i) at 1245 cm⁻¹ vanished and was replaced by two weaker bands (**Figure 3**A.h and i)  at 1265 and 1225 cm⁻¹. The gradual reduction in intensity of these new peaks with longer treatment times indicates the progressive disruption and solubilization of guaiacyl lignin units responsible for Guaiacyl unit stretching vibrations (C-C, C-O, C=O), which gave a cumulative signal at 1245 cm⁻¹. However, the peak responsible for the syringyl unit stretching at 1325 cm⁻¹ (**Figure 3**C.g) remains the same without changing the peak area, while the peak area for the guaiacyl region decreases due to the partial removal of lignin.^11^  Overall, these spectral changes confirm that the NaOH/Na₂SO₃ treatment effectively removed hemicellulose and degraded lignin structures, while preserving the cellulose framework. Overall functional group assignments are given in the supplementary table 5.

The FTIR spectra of graphene-coated wood samples compared with their corresponding delignified counterparts are shown in **Figure 3**D. Coating with graphene ink produced several characteristic changes in the FTIR spectra. For example, in the ‘l’ region (3285–3020 cm⁻¹), all coated samples exhibited a broadened O–H envelope. This region corresponds to hydroxyl stretching vibrations, and the broadening reflects stronger and more heterogeneous hydrogen bonding after removal of hemicellulose and lignin, exposed cellulose hydroxyls, and increased surface area, allowing new interfacial interactions (intermolecular) with the graphene coating.^5,12^ Additional broadening was observed in the ‘o’ region (1905–1650 cm⁻¹), arising from overlapping contributions of bound water bending, aromatic C=C vibrations, and carbonyl groups.^13^ In the more extensively delignified samples (DW-20 and DW-25), a distinct broadening also appeared in the ‘p’ region (750–450 cm⁻¹), amplified by higher ink uptake in these porous substrates. Notably, the intensities of cellulose marker bands at 1165–885 cm⁻¹ (C–O–C, C–O, and β-glycosidic linkages) decreased markedly after coating.^7,8^ This attenuation is attributed to the graphene overlayer screening the ATR evanescent field and partially masking cellulose signals, providing evidence for successful surface compounding of graphene on the delignified wood. FTIR analysis was also performed to compare the composition of the graphene dispersion and its dried form, with particular attention to identifying surfactant-related features (region q) (**Figure S**17).

**Supplementary Discussion 3: Mechanical properties**

The cellulose component of wood provides mechanical strength while the surrounding lignin matrix transfers load to cellulose.^14^ Under mechanical stress, resistance to deformation results from breaking and reformation of hydrogen bonds from cellulose fibril sliding and cellulose-hemicellulose interactions. Hydrogen bonding is maximized when the volume fraction of load-bearing cellulose is maximized. Partial delignification followed by densification retains the alignment of cellulose nanofibrils, but much more densely packed than NW (Figure 2A and Figure 2B). This leads to a reduction in volume and the collapse of lumina (Figure 2J and Figure 3E). The collapse of lumina results in a greater volume fraction of load-bearing cellulose and hence maximized hydrogen bonding, hence an improvement of mechanical properties.^14^ Additionally, Graphene’s aromatic sp² carbon domains can interact with the hydrophobic faces of nanocellulose via π–π stacking and CH–π interactions - these non-covalent interactions further stabilize the composite and facilitate stress transfer.^15^

# **Characterization Methods**

The microstructures of the samples were observed by scanning electron microscope (SEM) (JEOL JSM-7610F, UAE). The tangential and cross-sectional surfaces of all samples were sputtered with a thin Au layer for 15 seconds. The cross sections of the samples were prepared as follows; All samples were stabilized in tert-butanol (TBA) for 1 hour to preserve the integrity of the wet nanonetwork in the dry state. The samples were then snap-frozen in liquid nitrogen and subjected to lyophilization (24 hours, Christ Alpha 1-2 LD Lyophilizer) to obtain dry, structurally intact architectures. The chemical components of the GDW samples were investigated by Raman spectroscopy. Raman measurements were performed using a confocal Raman microscope, which employed a 532 nm laser for excitation at 2.33 eV and a 50x objective lens, with the laser power meticulously maintained below 1 mW to prevent sample damage. FTIR spectra were recorded using a Bruker Vertex 80v spectrometer equipped with an ATR crystal (A225-Platinum) to analyze the chemical structure and functional group changes in delignified wood before and after graphene coating. The measurements were carried out at a resolution of 4 cm⁻¹ with 64 scans collected for both the sample and background in the range of 4000–400 cm^-1^.  The tensile properties of the samples were measured using an Instron 5565 universal tester with a 50 kN load cell at room temperature (25 ± 4 °C) and 45–60% RH. The dimensions of the tensile samples were approximately 100 *mm ×* 5 *mm ×* 1.5-4 *mm* (length *×* width *×* thickness). The samples were clamped at both ends and stretched along the wood fiber direction with a constant test speed of 5 *mm*/*min*. UV-vis-NIR analysis was carried out using a Lambda 750S (PerkinElmer) spectrometer. To ensure accurate results, an integrating sphere was employed. The UV-vis-NIR measurements covered a wavelength range of 200 to 2,500 nm. For transmittance measurements, the sample was set in front of an input port of the integrating sphere, a light source was applied as the incident beam, and the output light was collected through a detector place on the output port of the integrating sphere. For total reflection measurements, the sample was placed on the output port ooposite to the incident light from the input port, and the reflected light was collected by a detector on the side of the integrating sphere. A 3D optical surface profilometer was used to measure the ablated depth and crater area through intensity-based segregation. The determination of porosity and cell wall thicknesses from SEM images was conducted using the Fiji-ImageJ software version 2.9.0 (Rasband, 1997). Void regions were highlighted, and the area occupied by them was measured. Porosity was computed by dividing the void area by the total area of the wood sample within the scanning electron microscopy (SEM) images. The thermal diffusivity (α) and specific heat capacity (C_p_) were measured by the laser flash apparatus (LFA, NETZSCH 467, Germany) at 25 °C. The thermal conductivity (k) was calculated as k = α × C_p_ × ρ, where α and ρ are the thermal diffusivity and the density of the samples, respectively. Sample mass was measured using an analytical balance with a resolution of ${10}^{-4}$g. Sample dimensions were measured using a digital caliper with a resolution of $0.01$mm.

# **Materials and Experimental Setup**

# **Chemicals:**

Balsa wood (Ochroma pyramidale) was purchased from Creative Minds (Abu Dhabi, UAE), Graphite flakes (average size of 60-90 µm), Perchloric acid (*HClO*_4_, 70 wt.%), Sodium sulfite (*Na*_2_*SO*_3_, 98%, and sodium hydroxide (NaOH, 98%) were purchased from Sigma-Aldrich (Abu Dhabi, UAE). Sodium Deoxycholate (SDC, *C_24_H_39_NaO_4_*, 97%) was purchased from bioWORLD (Dubai, UAE). All chemicals were directly used without further purification. Deionized (DI) water was used throughout the experiment.

# **Synthesis Procedures:**

*Synthesis of graphene dispersion.* Graphene was synthesized according to the method reported previously.^2^ 2.5 mL of Perchloric acid was added to 1 g of graphite flakes and heated for 30 mins at 200 °C to form intercalated graphite (IG). IG was then subjected to microwave irradiation for ~1 min, forming thermally expanded graphite (EG). EG was then added to a solution of SDC (10 %weight) in DI water, and the mixture was exfoliated using a high-pressure homogenizer (HPH) for 40 cycles at a pressure of 1000 mbar, forming a graphene dispersion.

*Synthesis of delignified and graphene delignified wood.* To prepare delignified wood (DW), natural wood (NW) samples measuring 17 cm *×* 7.6 cm *×* 4 mm (length *×* width *×* thickness) with a density of 0.18 g cm^-3^ were partially delignified according to previous literature.^16^ The NW samples were treated with a boiling aqueous solution of 2.5 M NaOH and 0.4 M *Na*_2_*SO*_3_ for *x* hours, followed by immersion in deionized (DI) water multiple times to remove residual chemicals. Next, the partially delignified wood was oven-dried at a temperature of 60 °C for 12 hours. The dried DW is also referred to as “shrunken wood”. The shrunken wood was then immersed in graphene dispersion (i.e., the "graphene-shocking" process) to form moldable/flexible wood. This wood oven-dried again under flat aluminium plates at 100 °C for 12 hrs. The samples are denoted as GDW-x, where x corresponds to the delignification duration in hours.

# **Propulsion Tests:**

The laser setup illustrated in **Figure 4**A was employed to execute all laser propulsion tests on the wood samples. It consists of a 450 nm continuous wave (CW) laser diode, 3D-printed housing for the laser, and a 120 fps camera. The setup is placed inside a thermal vacuum chamber (TVAC). Wood samples weighing 130±0.003 mg are hung by a carbon fiber thread at the focal point of the laser diode. Pressure level inside the TVAC and laser power are set using an external controller. The camera is turned on before the laser is turned on, and the propulsive motion is recorded for analysis. Note that the laser current is varied from 0.5 A to 3 A, corresponding to a power varied from 0.42 to 5.40 W as per the power meter readings. With an ablation spot diameter of 1 mm on the samples at the focal point, the corresponding intensity on the samples is varied from 0.54 x 10^6^ to 6.88 x 10^8^ W m^-2^.

To quantify the displacement in pendulum motion, the geometric boundaries of the samples were detected and used to calculate the geometric centers’ x and y coordinates in pixels in each frame. To track the motion of the object, the SeqTrack tracker is used, which is built on a simple encoder-decoder transformer architecture.^17^ As shown in **Figure S**6, the tracker begins with a template image of the object, which serves as the reference for the tracking process. Each subsequent video frame is treated as a search image, where the network attempts to locate the object. Both the template and search images are divided into small regions, which are converted into numerical representations called embeddings. These embeddings encode visual features such as brightness, texture, and edges, enabling the network to compare the template with the search frames and find the best match, even if the object moves or changes slightly in appearance. In SeqTrack, the object’s bounding box is represented as a sequence of discrete tokens corresponding to its position and size ([x, y, w, h]). The encoder extracts visual features from the video frames, while the decoder autoregressively generates the bounding box tokens based on these features. Two special tokens, start and end, indicate the beginning and completion of the sequence generation. During inference, the decoder starts with the start token and predicts each bounding box value in sequence, appending each new token until the full bounding box is generated. The predicted bounding box is then recorded, and its center is used as the object’s position in that frame. By repeating this process across all frames, SeqTrack produces a continuous trajectory of the object’s motion. Finally, the recorded positions are converted into displacement values along the X and Y directions, and the angular displacement (θ) is calculated to provide a complete description of the sample’s oscillatory motion.

# **Calculations**

# **Sample density:**

The density of each specimen ($\rho$) was measured from its measured mass and geometric volume:

$$\begin{aligned} \rho=\frac{m}{V} \#\left( 1 \right) \end{aligned}$$

Where $m$ is the mass of the sample and $V$ is the volume. The mass was measured using an analytical balance with a resolution of ${10}^{-4}$g. The samples had a rectangular geometry, and their volume was calculated as:

$$\begin{aligned} V=l\times w\times t\#\left( 2 \right) \end{aligned}$$

Where $l$, $w$, and $t$ represent the length, width, and thickness of the specimen, respectively. These dimensions were measured using a digital caliper with a resolution of $0.01$mm.

# **Propulsion Parameters:**

The generated thrust force (*F*) is calculated according to the following equation for a hanging pendulum setup(Polk et al., 2017) :

$$\begin{aligned} I\ddot{\theta}+c\dot{\theta}+k\theta=F\left( t \right)L \#\left( 3 \right) \end{aligned}$$

where: *θ*: is the angular displacement of the sample, *I* is the moment of inertia of the pendulum bob, *c* is the damping coefficient, *k* is the effective spring constant, defined as:

$$\begin{aligned} k=k_{s}+mgL\#\left( 4 \right) \end{aligned}$$

where *k_s_* is the additional spring constant (e.g., due to the stiffness of the suspension system), *m* is the mass of the pendulum bob, *g* is the acceleration due to gravity, and *L* is the pendulum length. The damping coefficient *c* and the spring constant *k* are estimated by minimizing the residuals between the observed and theoretical behavior of the pendulum when the laser is turned off, i.e. thrust force is zero:

$$\begin{aligned} R= \sum_{i=1}^{N} {I \ddot{\theta_{i}}+c\dot{\theta_{i}}+k\theta_{i}}^{2}\#\left( 5 \right) \end{aligned}$$

where $R$ is the sum of squared residuals, and $\ddot{\theta_{i}}$, $\dot{\theta_{i}}$, and $\theta_{i}$ are the observed angular acceleration, velocity, and displacement at the *i*-th time step. The specific impulse (*I_sp_*) is calculated using the relationship:

$$\begin{aligned} I_{sp}= \frac{F}{\dot{m}g_{0}}\#\left( 6 \right) \end{aligned}$$

Where *F* is the calculated thrust force, $\dot{m}$ is the mass flow rate of the material being ablated, and $g_{0}$ = 9.81 m s^-1^ is the standard acceleration due to gravity. the total mass loss was obtained from direct gravimetric measurements, where each sample was weighed before and after laser irradiation using a precision analytical balance. The mass loss ($\Delta m$) was therefore calculated as

$$\begin{aligned} \Delta m=m_{before}- m_{after}\#\left( 7 \right) \end{aligned}$$

This approach avoids uncertainties associated with irregular ablation crater geometry and ensures that the measured mass loss reflects the total material removed during laser irradiation. The propellant mass consumption rate ($\dot{m}$) was then determined as

$$\begin{aligned} \dot{m}= \frac{\Delta m}{\Delta t_{irr}} \#\left( 8 \right) \end{aligned}$$

Where $\Delta t_{irr}$ is the total irradiation time obtained from video frames in which the laser is ON. The momentum coupling coefficient (C_m_) is calculated as follows,

$$\begin{aligned} C_{m}= \frac{F}{P}\#\left( 9 \right) \end{aligned}$$

Where P is the laser power. To investigate the target’s surface morphology following laser interaction, all targets were subjected to 3 seconds of laser irradiation with a wavelength of 450 nm and a power measured to be 4.492 W using a power meter, with a spot size of 1 mm. The ablation efficiency ($\eta$) is the ratio of total kinetic energy generated by the target and the laser input energy, calculated as:

$$\begin{aligned} \eta=\frac{1}{2}C_{m}I_{sp}g \#\left( 10 \right) \end{aligned}$$

# **Attenuation coefficient:**

For anisotropic materials with scattering, such as wood composites, the attenuation coefficient ($\propto$) can be calculated from the total transmittance ($T_{tot}$) as follows,^19,20^

$$\begin{aligned} T_{tot}= e^{-\propto d}\#\left( 11 \right) \end{aligned}$$

Where $d$ is sample thickness. This model has the same form as the Beer-Lambert law but it depends on diffusion and absorption coefficient, unlike the Beer-Lambert law valid only for non-scattering materials.

# **Uncertainty and error propagation:**

The uncertainty of mass measurement is calculated as

$$\begin{aligned} \sigma_{\Delta m}= \sqrt{\sigma_{m_{before}}^{2}+\sigma_{m_{after}}^{2}} =\sqrt{2}\sigma_{m}\#\left( 12 \right) \end{aligned}$$

Where $\sigma_{m}$ = 1 x 10^-4^ g in this work. $\Delta t_{irr}$ is counted from video frames with frame rate $f_{ps}$ and we assume a 4-frame uncertainty in the ON duration,

$$\begin{aligned} \sigma_{\Delta t}= \frac{1}{f_{ps}} \#\left( 13 \right) \end{aligned}$$

Then the error in mass loss rate is calculated using first-order Gaussian propagation:

$$\begin{aligned} \sigma_{\dot{m}}^{2}= \left( \frac{\partial\dot{m}}{\partial(\Delta m)}\sigma_{\Delta m} \right)^{2}+ \left( \frac{\partial\dot{m}}{\partial(\Delta t_{irr})}\sigma_{\Delta t} \right)^{2}\#\left( 14 \right) \end{aligned}$$

The derivatives are

$$\begin{aligned} \frac{\partial\dot{m}}{\partial(\Delta m)}= \frac{1}{\Delta t_{irr}} , \frac{\partial\dot{m}}{\partial(\Delta t_{irr})}= -\frac{\Delta m}{{\Delta t}_{irr}^{2}}= -\frac{\dot{m}}{\Delta t_{irr}}\#\left( 15 \right) \end{aligned}$$

Thus,

$$\begin{aligned} \sigma_{\dot{m}}= \sqrt{\left( \frac{\sigma_{\Delta m}}{\Delta t_{irr}} \right)^{2}+ \left( \frac{\dot{m}\sigma_{\Delta t}}{\Delta t_{irr}} \right)^{2}}\#\left( 16 \right) \end{aligned}$$

The calculation of propagation error for propulsion metrics ($C_{m}$, $I_{sp}$, and $\eta$) requires the calculation of the uncertainty in thrust measurement arising from the optical tracking and pendulum dynamics used to extract the thrust signal. Numerical differentiation errors were propagated through the pendulum equation of motion to determine the uncertainty in the inferred thrust force.

Thrust is calculated from the pendulum equation of motion using the tracked bob angle $\theta(t)$:

$$\begin{aligned} \frac{I\ddot{\theta}\left( t \right)+c\dot{\theta}+(k_{s}+mgL)\theta\left( t \right)}{L} =F(t)\#\left( 17 \right) \end{aligned}$$

Where $I=mL^{2}$is the moment of inertia of the specimen-pendulum system treated as a bob, $L$ is the string length, $c$ is the damping coefficient, $k_{s}$ is the torsional spring term, and $g$ is the gravitational acceleration 9.81 m s^-2^. The AI tracker outputs centroid coordinates (x,y). A calibration grid provides the pixel-to-meter scale ($s$) in meters/pixel. The lateral displacement in meters is $x\left( t \right)=sx_{px}(t)$. The pendulum angle is obtained from the geometric relation

$$\begin{aligned} \theta\left( t \right)=\sin^{-1} \left( \frac{x\left( t \right)}{L} \right)\approx\frac{x\left( t \right)}{L}\#\left( 18 \right) \end{aligned}$$

The displacement uncertainty from tracking is:

$$\begin{aligned} \sigma_{x}=5\times{10}^{-5}\#\left( 19 \right) \end{aligned}$$

The angular uncertainty becomes

$$\begin{aligned} \sigma_{\theta}=s\frac{\sigma_{x}}{L}\#\left( 20 \right) \end{aligned}$$

Angular velocity and acceleration are obtained through numerical differentiation with sampling interval $\Delta t=1/f_{ps}$. Finite difference derivatives have an uncertainty of:

$$\begin{aligned} \sigma_{\dot{\theta}}\approx\frac{\sigma_{\theta}}{\Delta t} , \sigma_{\ddot{\theta}}\approx\frac{\sqrt{6}\sigma_{\theta}}{{\Delta t}^{2}}\#\left( 21 \right) \end{aligned}$$

Treating $I$, $c$, $k_{s}$, $m$, and $L$ as fixed parameters, the dominant measurement uncertainty originates from tracking noise propagated through $\theta$,$\dot{\theta}$, and$\ddot{\theta}$. Applying first-order gaussian error propagation to equation (17) yields

$$\begin{aligned} \sigma_{F}= \sqrt{\left( \frac{I}{L} \sigma_{\ddot{\theta}} \right)^{2}+ \left( \frac{c}{L}\sigma_{\dot{\theta}} \right)^{2}+ \left( \frac{k_{s}+mgL}{L}\sigma_{\theta} \right)^{2}}\#\left( 22 \right) \end{aligned}$$

Where $\sigma_{\theta}$, $\sigma_{\dot{\theta}}$, and $\sigma_{\ddot{\theta}}$ are obtained from equations (12) and (13). Specific impulse is calculated from thrust and mass flow rate as

$$\begin{aligned} I_{sp}=\frac{F}{\dot{m}g}\#\left( 23 \right) \end{aligned}$$

Applying first-order Gaussian propagation gives

$$\begin{aligned} \sigma_{I_{sp}}= \sqrt{\left( \frac{\sigma_{F}}{\dot{m}g} \right)^{2}+ \left( \frac{F \sigma_{\dot{m}}}{\dot{m}^{2}g} \right)^{2}}\#\left( 24 \right) \end{aligned}$$

The momentum coupling coefficient is defined as

$$\begin{aligned} C_{m}=\frac{F}{P}\#\left( 25 \right) \end{aligned}$$

Where $P$ is the incident laser power,

$$\begin{aligned} \sigma_{C_{m}}=\frac{\sigma_{F}}{P}\#\left( 26 \right) \end{aligned}$$

Finally, the ablation efficiency is defined as

$$\begin{aligned} \eta=\frac{1}{2}C_{m}I_{sp}g \#\left( 27 \right) \end{aligned}$$

Applying first-order Gaussian propagation yields

$$\begin{aligned} \sigma_{\eta}= \sqrt{\left( \frac{1}{2}I_{sp}g\sigma_{C_{m}} \right)^{2}+ \left( \frac{1}{2}C_{m}g\sigma_{I_{sp}} \right)^{2}}\#\left( 28 \right) \end{aligned}$$

For example, for one of the experiments on a NW specimen at an intensity of 5.72 MW m^-2^:

$$\begin{aligned} \sigma_{\dot{m}}= \sqrt{\left( \frac{\sigma_{\Delta m}}{\Delta t_{irr}} \right)^{2}+ \left( \frac{\dot{m}\sigma_{\Delta t}}{\Delta t_{irr}} \right)^{2}} \\ = \sqrt{\left( \frac{1.414\times{10}^{-7}}{187.880} \right)^{2}+ \left( \frac{2.661\times{10}^{-8}\times0.033}{187.880} \right)^{2}}\#\left( 29.1 \right) \\ = 7.527\times{10}^{-10} kg/s \end{aligned}$$

$$\begin{aligned} \sigma_{F}= \sqrt{\left( \frac{I}{L} \sigma_{\ddot{\theta}} \right)^{2}+ \left( \frac{c}{L}\sigma_{\dot{\theta}} \right)^{2}+ \left( \frac{k_{s}+mgL}{L}\sigma_{\theta} \right)^{2}} \\ =\sqrt{\left( \frac{7.948\times{10}^{-9}}{0.0225} 0.980 \right)^{2}+ \left( \frac{7.340\times{10}^{-6}}{0.0225}0.0133 \right)^{2}+ \left( \frac{3.465\times{10}^{-6}}{0.0225}4.444\times{10}^{-4} \right)^{2}}\#\left( 29.2 \right) \\ =4.364\times{10}^{-6} N \end{aligned}$$

$$\begin{aligned} \sigma_{I_{sp}}= \sqrt{\left( \frac{\sigma_{F}}{\dot{m}g} \right)^{2}+ \left( \frac{F \sigma_{\dot{m}}}{\dot{m}^{2}g} \right)^{2}} \\ = \sqrt{\left( \frac{4.364\times{10}^{-6}}{2.661\times{10}^{-8}\times9.81} \right)^{2}+ \left( \frac{0.238\times7.527\times{10}^{-10}}{{(2.661\times{10}^{-8})}^{2}\times9.81} \right)^{2}}\#\left( 29.3 \right) \\ =30.753 s \end{aligned}$$

$$\begin{aligned} \sigma_{C_{m}}=\frac{\sigma_{F}}{P} \\ =\frac{4.364\times{10}^{-6}}{4.492}\#\left( 29.4 \right) \\ =9.715\times{10}^{-7}N/W \end{aligned}$$

$$\begin{aligned} \sigma_{\eta}= \sqrt{\left( \frac{1}{2}I_{sp}g\sigma_{C_{m}} \right)^{2}+ \left( \frac{1}{2}C_{m}g\sigma_{I_{sp}} \right)^{2}} \\ = \sqrt{\left( \frac{1}{2}\times912.608\times9.81\times9.715\times{10}^{-7} \right)^{2}+ \left( \frac{1}{2}\times5.304\times{10}^{-5}\times9.81\times30.753 \right)^{2}}\#\left( 29.5 \right) \\ = 9.1 \times{10}^{-3}= 0.91\% \end{aligned}$$

The results for this specimen are:

$$\begin{aligned} \dot{m}= 2.661\times{10}^{-8} \pm7.527\times{10}^{-10} kg/s \left( relative 2.828\% \right) \\ F= 0.238 \pm0.004 mN (relative 1.832\%) \\ I_{sp}= 912.608 \pm30.753 s (relative 3.370\%)\#\left( 30 \right) \\ C_{m}= 53.040 \pm0.972 N/MW (relative 1.830\%) \\ \eta= 23.034 \pm0.91\% (relative 3.957\%) \end{aligned}$$

The uncertainty in density measurement is estimated using Gaussian propagation:

$$\begin{aligned} \frac{\sigma_{\rho}}{\rho}=\sqrt{\left( \frac{\sigma_{m}}{m} \right)^{2}+\left( \frac{\sigma_{l}}{l} \right)^{2}+\left( \frac{\sigma_{w}}{w} \right)^{2}+\left( \frac{\sigma_{t}}{t} \right)^{2}} \#\left( 31 \right) \end{aligned}$$

Where $\sigma_{m}=0.0001g$, $\sigma_{L}=\sigma_{W}=\sigma_{t}=0.01 mm$. For example, a typical NW sample with mass = 0.0150 g, L = W = 10 mm, and t = 4.6 mm, the relative uncertainty in density is estimated to be ~0.7%. Since density-specific $I_{sp}$ is $I_{sp}/\rho$, the uncertainty in density-specific $I_{sp}$ is propagated as

$$\begin{aligned} \frac{\sigma_{\frac{I_{sp}}{\rho}}}{\frac{I_{sp}}{\rho}}=\sqrt{\left( \frac{\sigma_{I_{sp}}}{I_{sp}} \right)^{2}+\left( \frac{\sigma_{\rho}}{\rho} \right)^{2}}\#\left( 32 \right) \end{aligned}$$

# **Cost of materials:**

The cost of NW and DW-25 is calculated based on the cost of natural balsa wood and chemicals used for delignification (Table S1). The cost of GDW-25 requires calculating the cost of producing 1 Kg of graphene, and the dosage of graphene per m^3^ of wood. The cost of producing graphene is the sum of the cost of energy input and the cost of chemicals.

*Energy demand for graphene production:* The total energy spent to intercalate graphite with, expand IG, and exfoliate EG is calculated through the amount of energy spent during the intercalation process using a hot plate MaXtir 500 H^21^, the expansion process using GMO1899 1100 W microwave oven^2^ and the exfoliation process using PSI-30 High pressure homogenizer^22^.

*HClO_4_ Intercalation Process:*

Power Input of Daihan hot plate MaXtir 500 H = 2300 watt per hour^21^

Power Input of Daihan hot plate MaXtir 500 H in 1 min = $\frac{Power input of MaXtir 500 H}{60 min}$

= $\frac{2300 watt}{60 min}$= 38.33 Watt/min

Total energy of Daihan hotplate MaXtir 500 H in 30 min = Power input in 1 min×30 min

= 38.33 Watt/min × 30 min

= 1149.9 Watt

The energy utilized during the intercalation process = 2.06 MJ

*Expansion Process:*

Power Input of the GMO1899 microwave oven = 1100 Watt per hour^2^

Power Input of the GMO1899 microwave oven in 1 min = $\frac{Power Input of GMO1899}{60 min}$

= $\frac{1100 watt}{60 min}$ = 18.33 Watt/min

Total Energy of the GMO1899 microwave oven in 30 min = 550 Watt

The energy utilized during the intercalation process = 0.99 MJ

*Exfoliation Process:*

Power Input of PSI-30 = 8.3 K watt per hour^22^

The energy utilized in the exfoliation process = 29.88 MJ

Total energy used during the entire process = 32.93 MJ

*Total cost of 1 Kg graphene:*

Cost of graphite = 6 USD per Kg4

Cost of HClO_4_ = 5 USD per Liter5

Cost of SDC = 285 USD per Kg^25^

Cost of energy utilized = (energy utilized during intercalation + energy utilized during expansion + energy utilized during exfoliation Process) × price for Watt of electricity in UAE (USD)6

= 9999.9 Watt hr × 0.00008 USD

= 0.799 USD

The total cost for producing 1Kg of Graphene = Cost of graphite + Cost of 2.5 L of HClO_4_ + Cost of %10 wt SDC + Cost of energy utilized

=6 USD + 2.5×5 USD + 285×0.1 USD + 0.799 USD

The total cost for producing 1Kg of Graphene = 47.799 USD

*Dosage of graphene per m^3^:*

The dosage of graphene per m^3^ = concentration of graphene dispersion × amount of

graphene dispersion used per piece of wood

= $\frac{20 g}{1 L}$ × $\frac{0.025 L}{5.3 {\times10}^{-5} m^{3}}$ = 9.37 Kg per m^3^

# **Supplementary Figures**


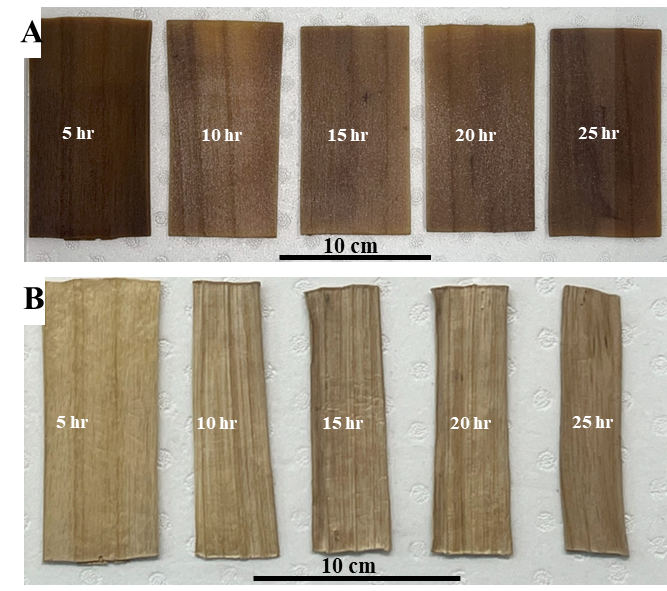


# **Figure S1.** **Digital images of the DW samples before and after drying.** A) DW-x. B) Shrunken wood.


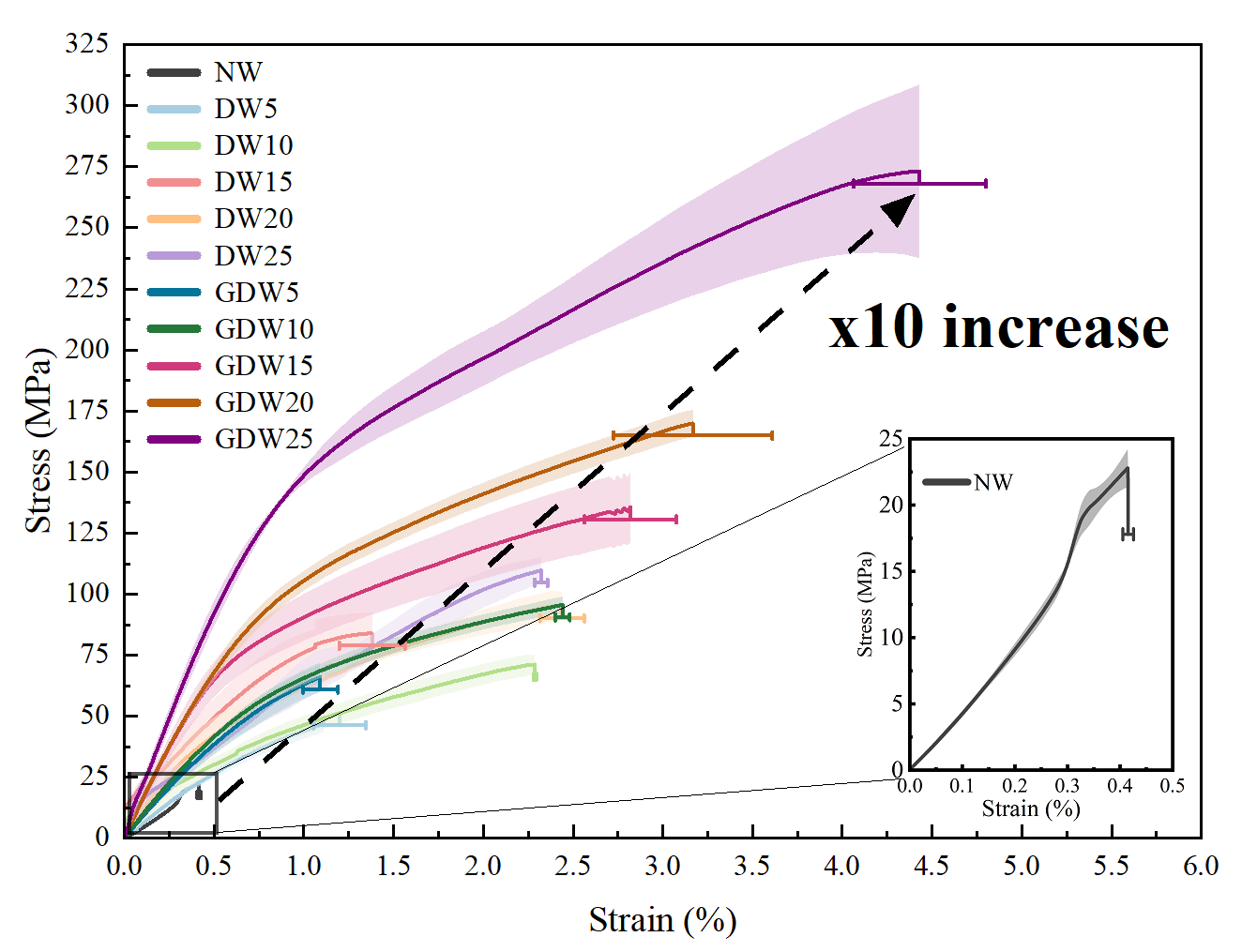


# **Figure S2**. **Tensile test results of NW, DW-x, and GDW-x composites.** The shaded region represents the standard deviation.


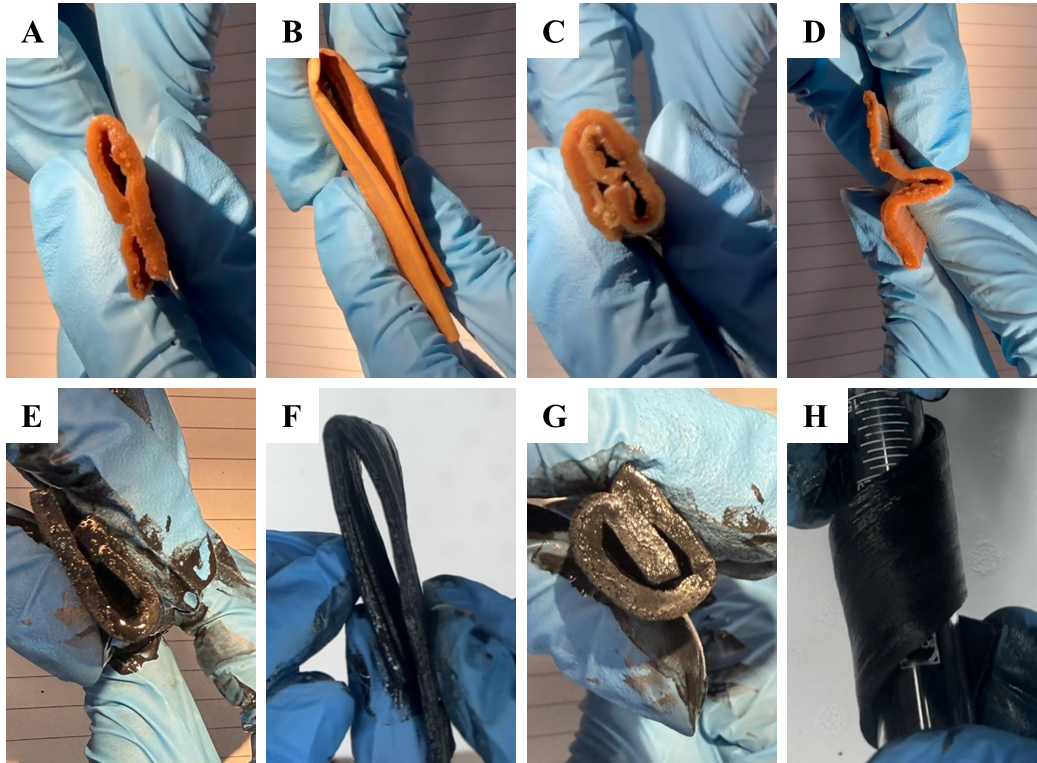


# **Figure S3.** **Digital images of MW samples post-shocking with DI water and graphene.** Samples folded by ~180° parallel to fiber direction, by ~180° perpendicular to fiber direction, into a spiral parallel to fiber direction, and into a random shape parallel to fiber direction of A-D) DW and E-H) GDW.


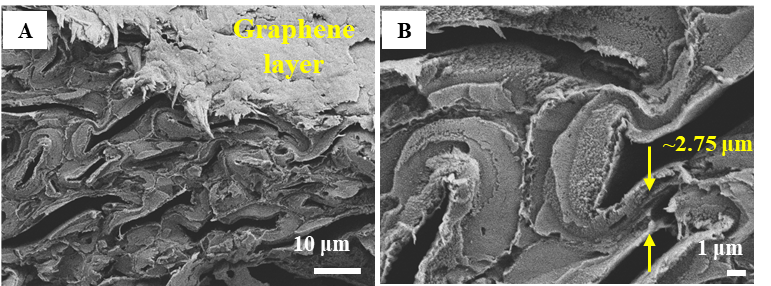


# **Figure S4.** **SEM images of GDW showing slit-shaped lumina region.** A) the graphene layer and “slit-shaped” lumina region with a porosity of 19% and B) cell wall thickness of ~2.75 μm.


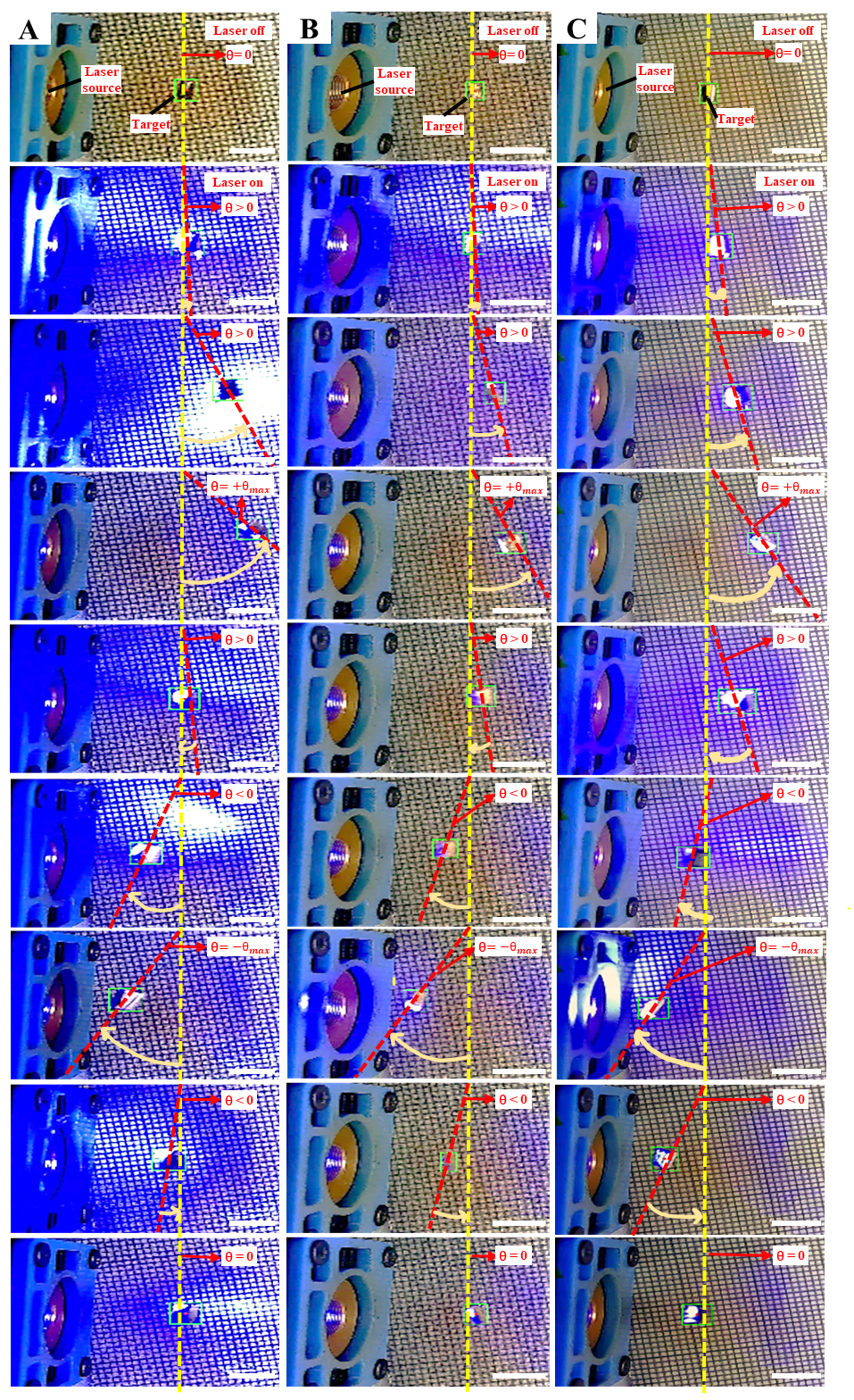


# **Figure S5. Digital images of the tracked sample movement in pendulum motion with time.** A) NW, B) DW, and C) GDW-25 targets.


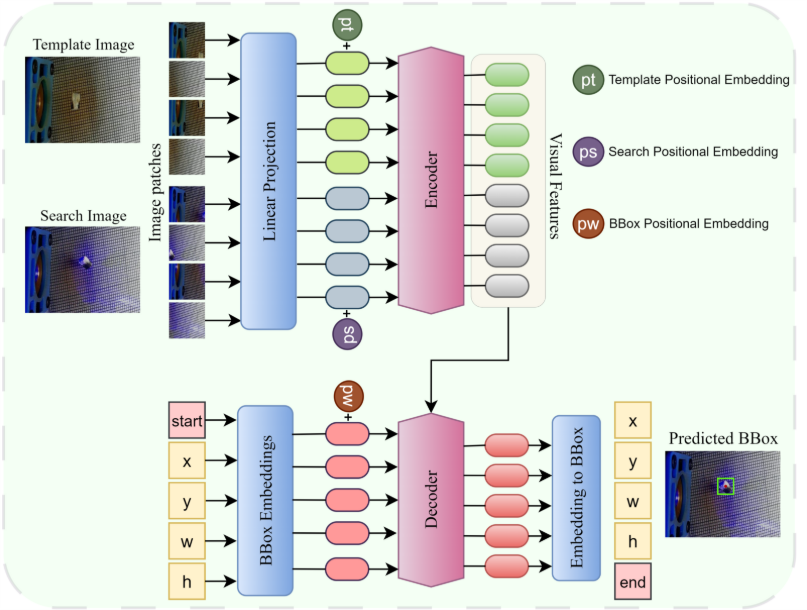


# **Figure S6. SeqTrack workflow.** The tracker uses a template image and processes each frame as a search image. Regions are converted into embeddings, and the encoder-decoder transformer predicts bounding boxes to generate the object’s continuous trajectory.


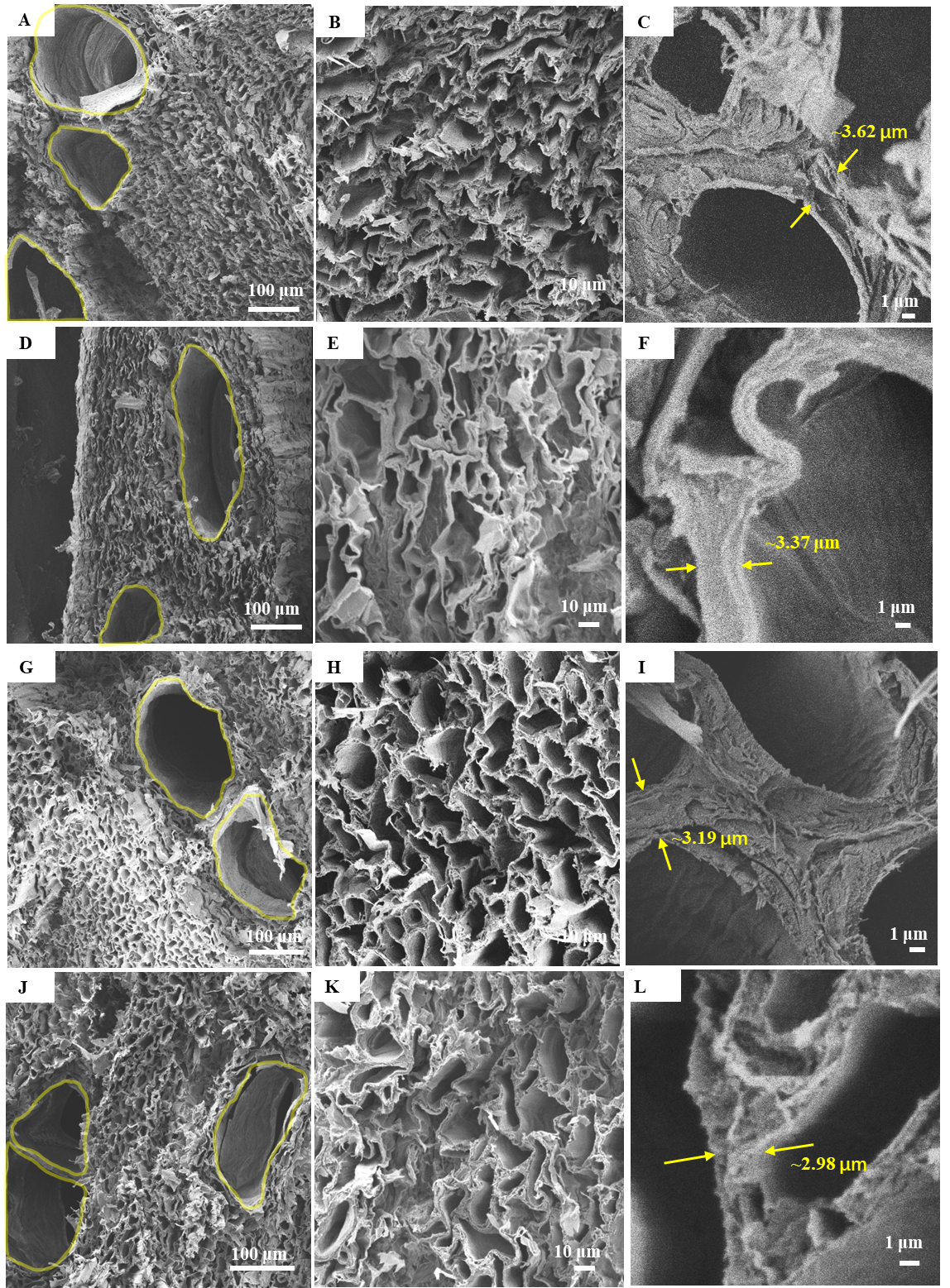


# **Figure S7.** **SEMs showing cross sections with vessels (highlighted in yellow), porosities of lumina, and cell wall thicknesses of different delignification times.** A-C) GDW5, D-F) GDW10, G-I) GDW15, and J-L) GDW20.


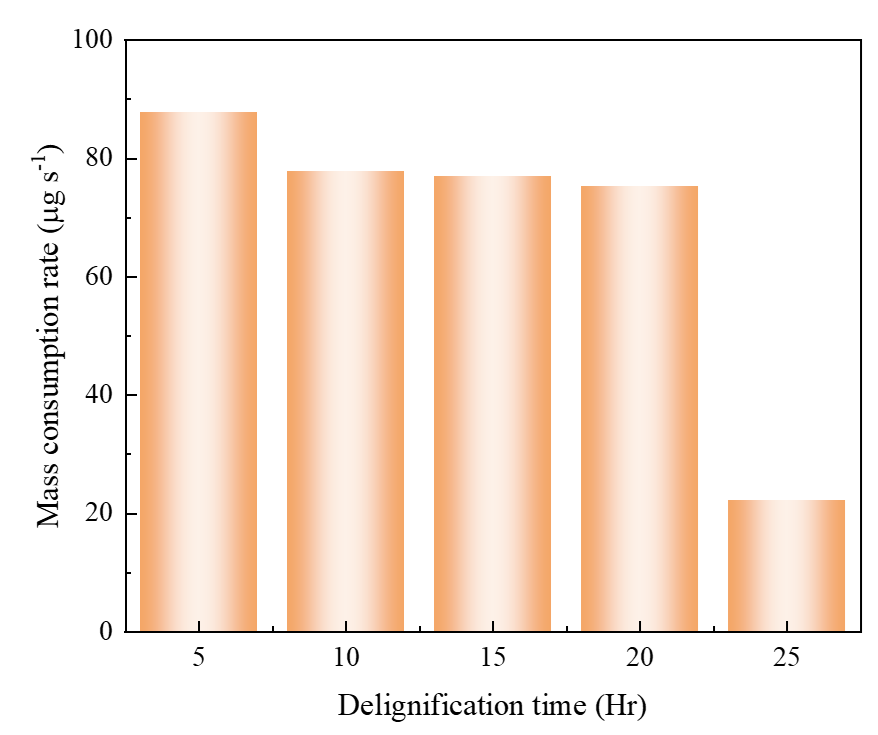


# **Figure S8. Mass loss rate vs delignification time for GDW-x composites.**

# **Figure S9. Ablation efficiency vs delignification time for GDW-x composites.**


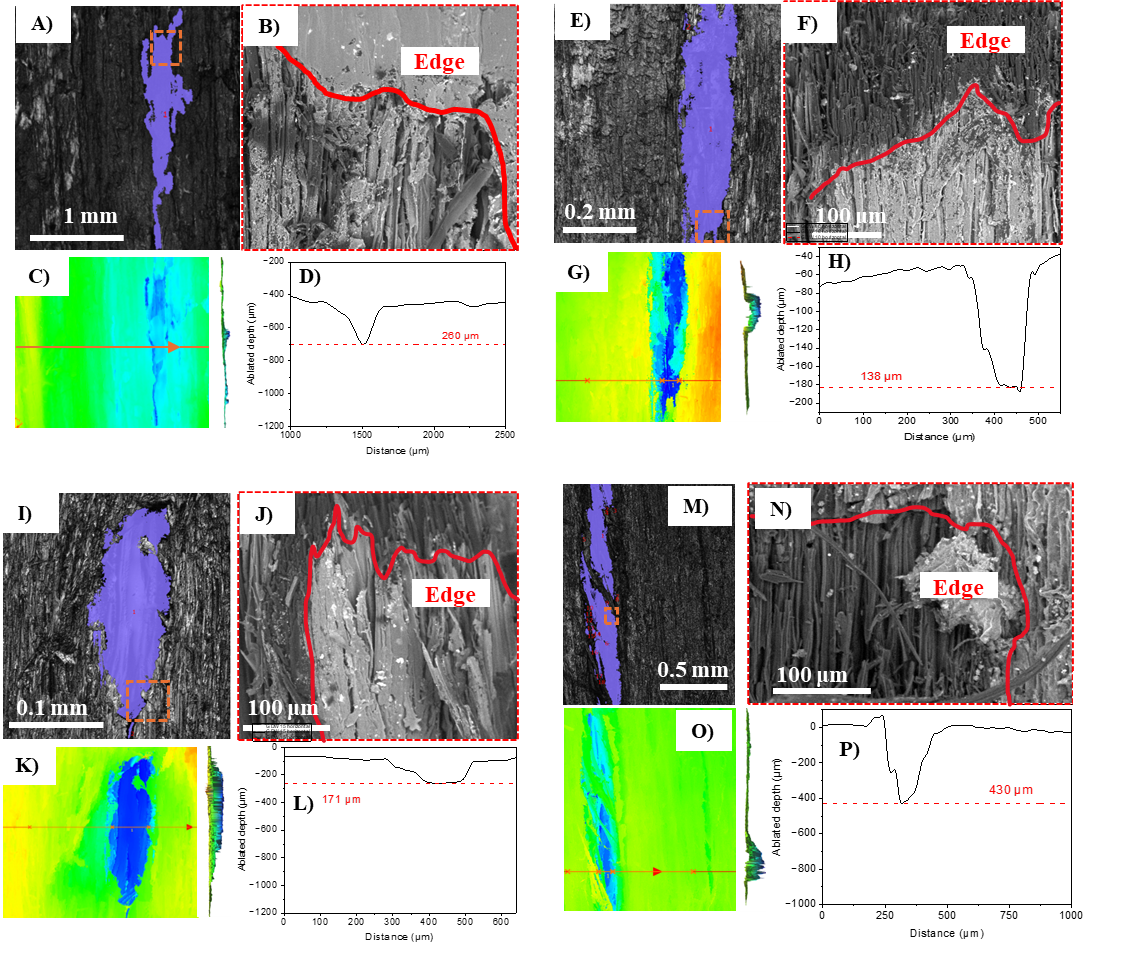


# **Figure S10. Crater area, edge, and depth analyses of GDW-x composites**. Profilometer images of ablated crater areas, SEM of ablated crater edges, and profilometer images of the crater depth of A-C) GDW-5, D-F) GDW-10, G-I) GDW-15, and J-L) GDW-20.


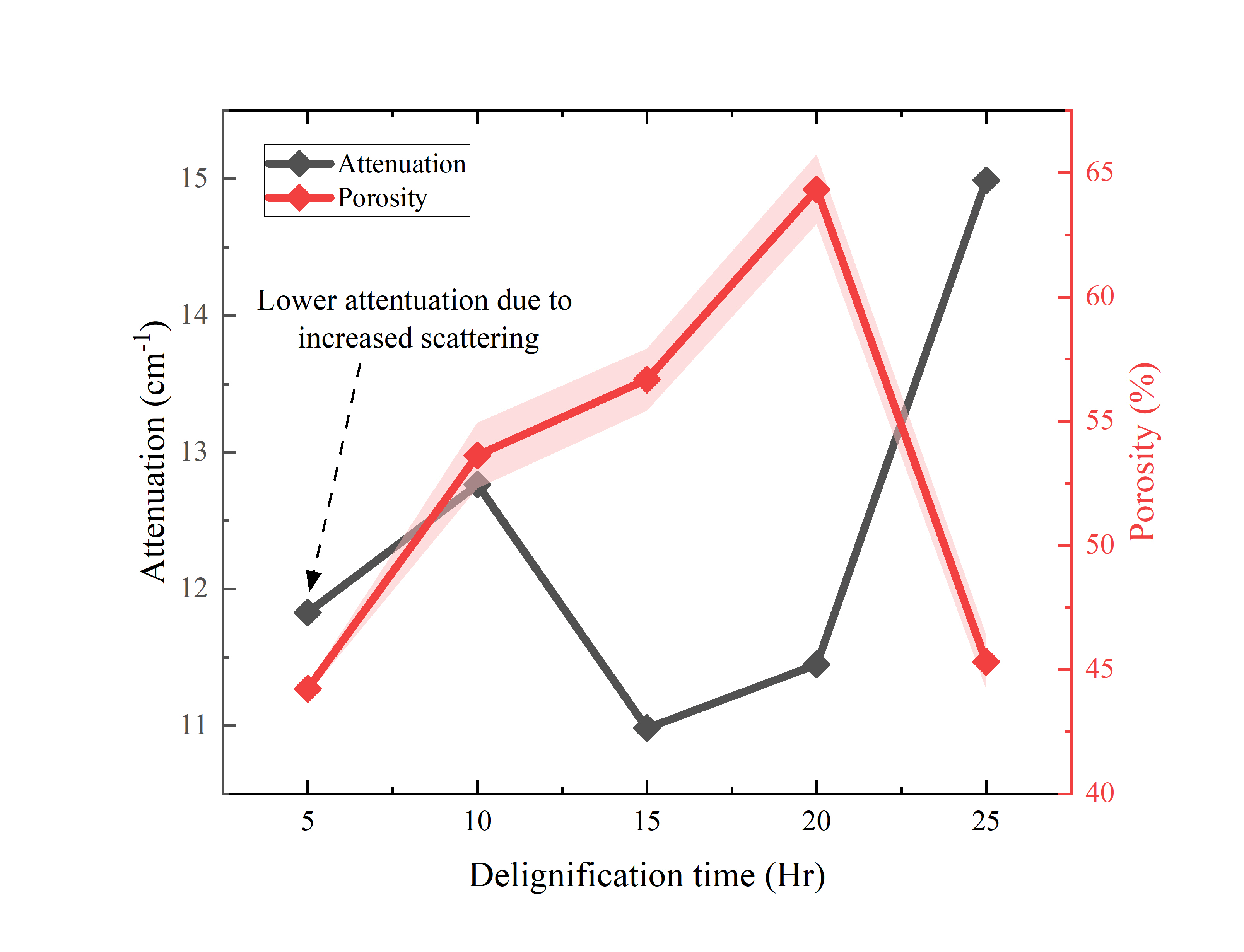


# **Figure S11. Attenuation coefficient and porosity for GDW-x composites.**

# **Figure S12. Ablation efficiency vs laser intensity for NW, DW-25, and GDW-25.**


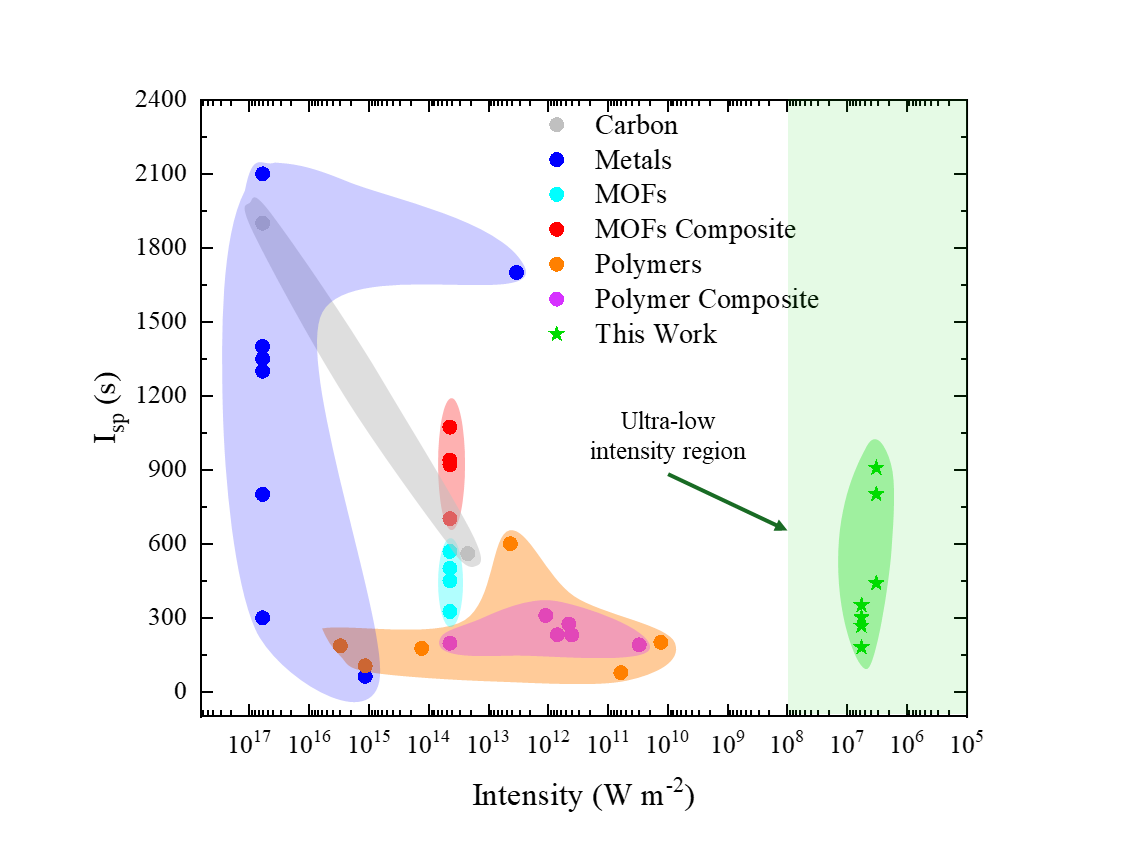


# **Figure S13. Comparison of I_sp_ and intensity with other material types.**


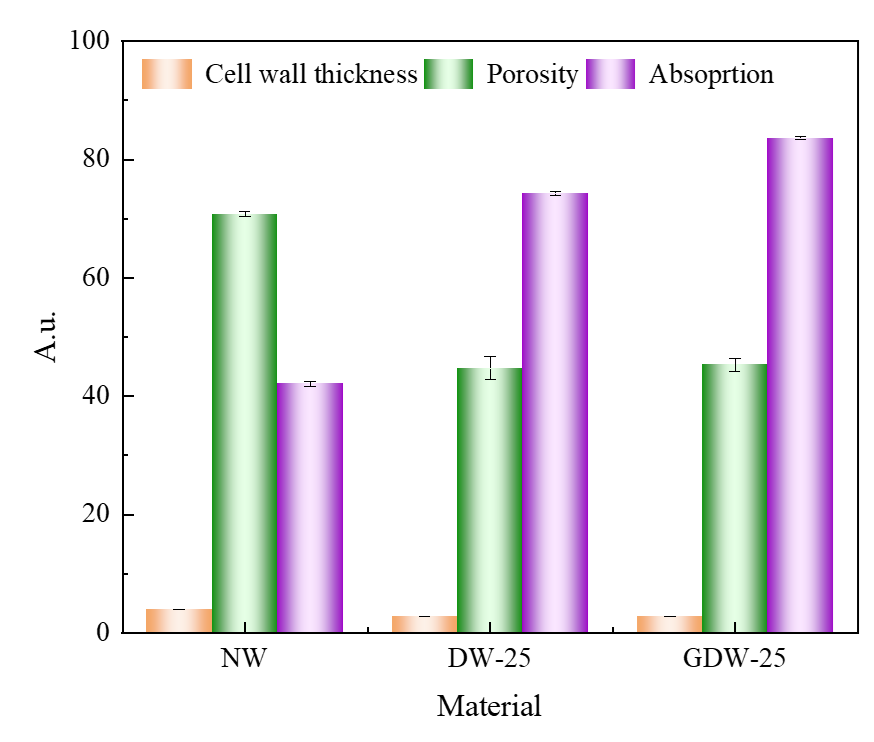


# **Figure S14. Comparison between microstructure and optical parameters of NW, DW-25, and GDW-25.**


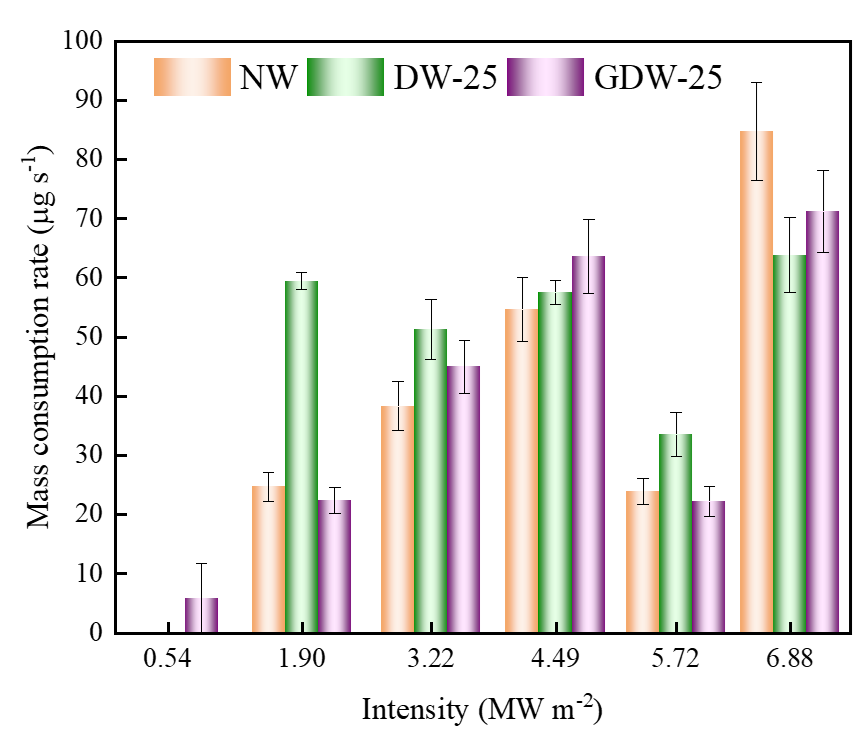


# **Figure S15. Mass consumption rate vs laser intensity for NW, DW-25, and GDW-25.**


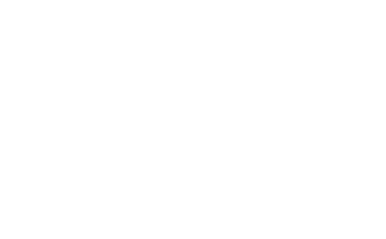


**B**

**A**

**C**

# **Figure S16. Propulsion parameters vs sample thickness.** A) NW, B) DW-25, and C) GDW-25.


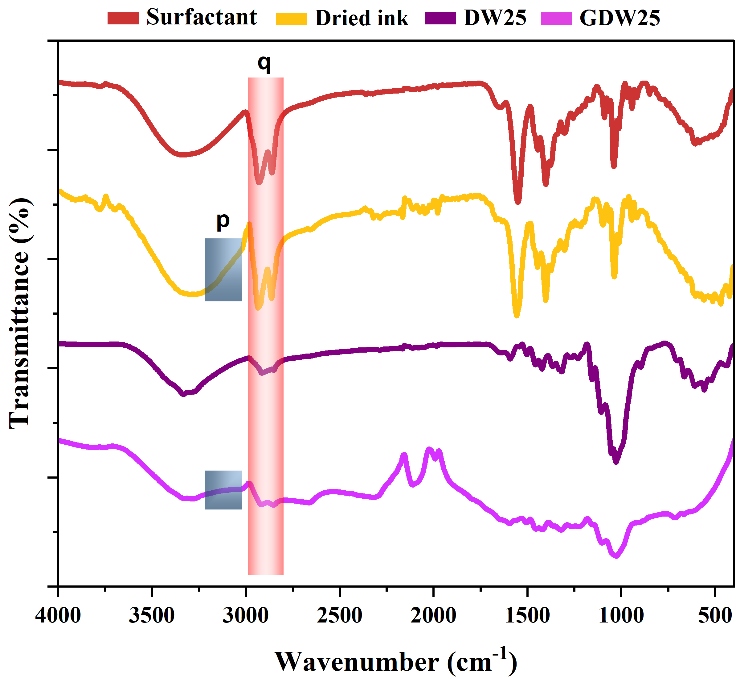


# **Figure S17. FTIR spectra of the surfactant used, dried graphene ink powder, delignified wood (DW-25), and graphene ink-coated delignified wood (GDW-25).**

# **Supplementary Tables**

# **Supplementary Table 1:** Cost of materials of NW, DW-25, GDW-25, Al 7075-T6, and PTFE.

| Material | Price | Dosage | Total cost ($/m^3^) |
| --- | --- | --- | --- |
| NaOH | 300 $/ton^16^ | 375 kg | 112.5 |
| Na_2_SO_3_ | 300 $/ton^16^ | 189 kg | 56.7 |
| NW | 384 $/m^3 27^ | 1 m^3^ | **384** |
| DW-25 (total) |  |  | 553.2 |
| Graphene | 47.80 $/Kg | 9.37 kg | **447.9** |
| GDW-25 (total) |  |  | **1001.1** |
| Al 7075-T6 | 4420 $/ton^28^ |  | 12410 |
| PTFE | 5.5 $/Kg^29^ |  | 12100 |

# **Supplementary Table 2:** The performance comparison of structural materials.

| Material | Threshold intensity  (MW m^-2^) | Density  (g cm^-3^) | Specific I_sp_  (s g^-1^ cm^-3^) | Specific tensile strength  (MPa g^-1^ cm^3^) |
| --- | --- | --- | --- | --- |
| NW  (this work) | **1.9** | 0.18±0.00126 | **5,043.00±188** | 126.03 |
| DW-25  (this work) | **1.9** | 0.50±0.0041 | 877.73±38.1 | 164.89 |
| GDW-25  (this work) | **0.54** | 0.51±0.0043 | **1,569.60±57.4** | **533.33** |
| Al | 4.44 x 10^6^ ^30^ | 2.7 | 630^31^ | 84-172^32^ |
| PTFE | 3.5 x 10^5^ ^33^ | 2.13 | 282^34^ | 6.57^35^ |

# **Supplementary Table 3:** The performance comparison of different propellants.

| Items | Material name | Material type | Density (g cm^-3^) | I_sp_ (s) | Specific I_sp_ (s g^-1^ cm^-3^) | Intensity (W m^-2^) | Reference |
| --- | --- | --- | --- | --- | --- | --- | --- |
| 1 | GDW20 | Wood composite | 0.79±0.007 | 315±22.79 | 444.30±29.06 | 5.72 x 10^6^ | This work |
| 2 | GDW15 | Wood composite | 0.60±0.0046 | 301±9.91 | 753.44±16.96 | 5.72 x 10^6^ | This work |
| 3 | GDW10 | Wood composite | 0.40±0.0037 | 267±17.56 | 668.34±44.32 | 5.72 x 10^6^ | This work |
| 4 | GDW5 | Wood composite | 0.58±0.005 | 180±8.71 | 310.34±15.26 | 5.72 x 10^6^ | This work |
| 5 | Graphite | carbon | 2.25 | 560 | 248.89 | 2.22 x 10^13^ | Zheng et al.^36^ |
| 6 | Carbon | carbon | 2.21 | 1900 | 859.73 | 6 x 10^16^ | Pakhomov et al.^37^ |
| 7 | Al | metal | 2.7 | 1700 | 629.63 | 3.37 x 10^12^ | Igarashi et al.^31^ |
| 8 | Al | Metal | 2.7 | 62.2 | 23.03 | 1.16 x 10^15^ | Jamil et al. ^38^ |
| 9 | Al | Metal | 2.7 | 2100 | 777.78 | 6 x 10^16^ | Jamil et al. ^38^ |
| 10 | Iron | Metal | 7.87 | 1400 | 177.89 | 6 x 10^16^ | Jamil et al. ^38^ |
| 11 | Cu | Netal | 8.96 | 1350 | 150.67 | 6 x 10^16^ | Jamil et al. ^38^ |
| 12 | Zinc | metal | 7.13 | 1300 | 182.33 | 6 x 10^16^ | Jamil et al. ^38^ |
| 13 | Tin | metal | 7.31 | 300 | 41.04 | 6 x 10^16^ | Jamil et al. ^38^ |
| 14 | Lead | metal | 11.43 | 800 | 70.00 | 6 x 10^16^ | Jamil et al. ^38^ |
| 15 | HKUST-1 | MOF | 1.312 | 568.43 | 433.25 | 4.41 x 10^13^ | Rao et al.^39^ |
| 16 | Cu-MOF-74 | MOF | 1.403 | 500 | 356.38 | 4.41 x 10^13^ | Rao et al.^39^ |
| 17 | Cu-MOF-2 | MOF | 1.444 | 450 | 311.63 | 4.41 x 10^13^ | Rao et al.^39^ |
| 18 | CPL-1 | MOF | 1.544 | 325 | 210.49 | 4.41 x 10^13^ | Rao et al.^39^ |
| 19 | GMM-HKUST-1 | MOF composite | 0.958 | 1072.94 | 1119.98 | 4.41 x 10^13^ | Rao et al.^40^ |
| 20 | GMM-(Cu-MOF-2) | MOF composite | 1.152 | 919.9 | 798.52 | 4.41 x 10^13^ | Rao et al.^40^ |
| 21 | GMM-(Cu-MOF-74) | MOF composite | 1.016 | 939.2 | 924.41 | 4.41 x 10^13^ | Rao et al.^40^ |
| 22 | GMM-(CPL-1) | MOF composite | 1.402 | 701.5 | 500.36 | 4.41 x 10^13^ | Rao et al.^40^ |
| 23 | PTFE | polymer | 2.13 | 600 | 281.69 | 4.3 x 10^12^ | Sinko et al.^34^ |
| 24 | PVC | polymer | 1.39 | 200 | 143.88 | 1.3 x 10^10^ | Phipps et al.^41^ |
| 25 | Polyurethane | polymer | 1.2 | 105.51 | 87.93 | 1.16 x 10^15^ | Jamil et al.^38^ |
| 26 | POM | Polymer | 1.43 | 77 | 53.85 | 6.06 x 10^10^ | Suzuki et al.^42^ |
| 27 | TP | polymer | 1.5 | 186 | 124 | 3 x 10^15^ | Lippert et al.^43^ |
| 28 | polymide film | polymer | 1.42 | 175 | 123.24 | 1.31 x 10^14^ | Zhang et al.^44^ |
| 29 | PLA:PDOF+0.5 rGO | Polymer composite | 1.24 | 309 | 249.19 | 1.1 x 10^12^ | Battocchio et al.^45^ |
| 30 | PLA:PDOF+0.25 rGO | Polymer composite | 1.24 | 231 | 186.29 | 7 x 10^11^ | Battocchio et al.^45^ |
| 31 | PLA:PDOF+2 rGO | Polymer composite | 1.24 | 274 | 220.97 | 4.5 x 10^11^ | Battocchio et al.^45^ |
| 32 | PLA:PDOF+1 rGO | Polymer composite | 1.24 | 230 | 185.48 | 4 x 10^11^ | Battocchio et al.^45^ |
| 33 | PVC+CNP | polymer composite | 1.38 | 196.35 | 142.28 | 4.4 x 10^13^ | Rao et al.^39^ |
| 34 | TP+C | polymer composite | 1.6 | 190 | 118.75 | 3 x 10^10^ | Phipps et al. ^46^ |

# **Supplementary Table 4:** Comparison between GDW and previous graphene materials' performance in light propulsion.

| Study | Material | Laser power | Pressure | Thrust  (μN) | C_m_  (N/MW) |
| --- | --- | --- | --- | --- | --- |
| Wang et al. (2020)^47^ | Graphene sponge | 70 mW | 3-5 Pa | 2 –4.3 | 28-62 |
| Gaudenzi et al. (2020)^48^ | Graphene on copper grid sail in microgravity | 0.1-1 W | 0.06-0.08 Pa | 8x10^-3^-0.248 | 0.08-0.25 |
| Wang et al. (2021)^49^ | Graphene sponge | 0.1 W | 0.002 – 6 Pa | Several μN | - |
| Wang et al. (2023)^50^ | Graphene on SiO_2_ | 85 mW | 5 Pa | 0.8 | 9.41 |
| Khattab et al. (2025)^51^ | Graphene aerogel | 3.5 W | 1.33 x 10^-3^ Pa | 36.6 | 10.46 |
| This work | GDW | 4.492 W | 3.33 x 10^-2^ Pa | **150–260±4** | 34-58±1.08 |

# **Supplementary Table 5:** Overall functional group assignments regarding cellulose, hemicellulose, and lignin components of wood.

|  | Wavenumber (cm^-1^) | | Functional group assignment | Wood component |
| --- | --- | --- | --- | --- |
|  | Reference | Observed |  |  |
| a | 3375 | 3400 | OH(3)-O(5) intra-molecular hydrogen bonding | Cellulose |
| b | 3340 | 3330 | Intra-molecular hydrogen bond of OH stretching |  |
| c | 3230 | 3270 | OH(6)-O(3) inter-molecular hydrogen bonding |  |
| d | 1730 | 1730 | C=O stretching | Hemicellulose |
| e | 1650 | 1654 | C=C stretching of the aromatic ring | Lignin |
| f | 1594 | 1590 | Aromatic ring vibrations |  |
| g | 1325 | 1325 | Syringyl unit C-O stretching |  |
| h | 1265 | 1265 | Guaiacyl unit C-O stretching |  |
| i | 1221 | 1224 | Guaiacyl unit stretching vibrations (C-C, C-O, C=O) |  |
| j | 1160 | 1150 | C-O-C asymmetric stretching | Cellulose |
| k | 1098 | 1103 | C-O stretching |  |

# **Supplementary References**

1. Ferrari, A. C. *et al.* Raman spectrum of graphene and graphene layers. *Phys. Rev. Lett.* **97**, (2006).

2. Shajahan, S., Elkaffas, R., Velusamy, D. B., Anjum, D. H. & Samad, Y. A. Sustainable Production of Graphene from Solar-Driven Expanded Graphite. *Advanced Energy and Sustainability Research* **6**, (2025).

3. Wu, W. *et al.* Fast chemical exfoliation of graphite to few-layer graphene with high quality and large size via a two-step microwave-assisted process. *Chemical Engineering Journal* **381**, (2020).

4. Ferrari, A. C. & Basko, D. M. Raman spectroscopy as a versatile tool for studying the properties of graphene. *Nat. Nanotechnol.* **8**, 235–246 (2013).

5. Djikanović, D. *et al.* Comparison of macromolecular interactions in the cell walls of hardwood, softwood and maize by fluorescence and FTIR spectroscopy, differential polarization laser scanning microscopy and X-ray diffraction. *Wood Sci. Technol.* **50**, 547–566 (2016).

6. Mvondo, R. R. N., Meukam, P., Jeong, J., Meneses, D. D. S. & Nkeng, E. G. Influence of water content on the mechanical and chemical properties of tropical wood species. *Results Phys.* **7**, 2096–2103 (2017).

7. Li, H., Lu, J. & Moa, J. Physiochemical lignocellulose modification by the formosan subterranean termite Coptotermes formosanus Shiraki (Isoptera: Rhinotermitidae) and its potential uses in the production of biofuels. *Bioresources* **7**, 675–685 (2012).

8. Shi, J., Xing, D. & Li, J. FTIR studies of the changes in wood chemistry from wood forming tissue under inclined treatment. *Energy Procedia* **16**, 758–762 (2012).

9. Ilyas, R. A., Sapuan, S. M. & Ishak, M. R. Isolation and characterization of nanocrystalline cellulose from sugar palm fibres (Arenga Pinnata). *Carbohydr. Polym.* **181**, 1038–1051 (2018).

10. Alqrinawi, H. *et al.* Effect of partial delignification and densification on chemical, morphological, and mechanical properties‏ of wood: Structural property evolution. *Ind. Crops Prod.* **213**, 118430 (2024).

11. Sammons, R. J. *et al.* Characterization of organosolv lignins using thermal and FT-IR spectroscopic analysis. *Bioresources* **8**, 2752–2767 (2013).

12. Kim, H. C., Panicker, P. S., Kim, D., Adil, S. & Kim, J. High-strength cellulose nanofiber/graphene oxide hybrid filament made by continuous processing and its humidity monitoring. *Sci. Rep.* **11**, 13611 (2021).

13. Calvini, P. & Gorassini, A. FTIR - Deconvolution spectra of paper documents. *Restaurator* **23**, 48–66 (2002).

14. Chen, C. *et al.* Structure–property–function relationships of natural and engineered wood. *Nat. Rev. Mater.* **5**, 642–666 (2020).

15. Kumar, S. & Saha, A. Graphene nanoplatelets/organic wood dust hybrid composites: physical, mechanical and thermal characterization. *Iranian Polymer Journal (English Edition)* **30**, 935–951 (2021).

16. Xiao, S. *et al.* Lightweight, strong, moldable wood via cell wall engineering as a sustainable structural material. *Science (1979).* **374**, 465–471 (2021).

17. Chen, X., Peng, H., Wang, D., Lu, H. & Hu, H. SeqTrack: Sequence to Sequence Learning for Visual Object Tracking. in *2023 IEEE/CVF Conference on Computer Vision and Pattern Recognition (CVPR)* 14572–14581 (2023). doi:10.1109/CVPR52729.2023.01400.

18. Polk, J. E. *et al.* Recommended practice for thrust measurement in electric propulsion testing. *J. Propuls. Power* **33**, 539–555 (2017).

19. Chen, H. *et al.* Green Nanotechnology of Cell Wall Swelling for Nanostructured Transparent Wood of High Optical Performance. *Small* **21**, 1–11 (2025).

20. Chen, H. *et al.* Thickness Dependence of Optical Transmittance of Transparent Wood: Chemical Modification Effects. *ACS Appl. Mater. Interfaces* **11**, 35451–35457 (2019).

21. https://najemalshahab.com/product/maxtir-500h-hi-temp-500%E2%84%83-digital-hotplates/.

22. https://www.sonics.com/site/assets/files/1165/vcx_750.pdf.

23. https://ceylongraphene.com.

24. https://dengzhuo.en.alibaba.com.

25. SDC price. https://www.alibaba.com/product-detail/Sodium-Deoxycholate-302-95-4-Surfactants_1600686008041.html?spm=a2700.7724857.0.0.54344c0cZcBT3d.

26. United Arab Emirates electricity prices, September 2023 | GlobalPetrolPrices.com.

27. Balsa wood. https://www.alibaba.com/product-detail/Paulownia-Balsa-Wood_62283182896.html?spm=a2700.galleryofferlist.normal_offer.d_title.714313a0EzF68Y.

28. Aluminium-7075-T6. https://www.alibaba.com/product-detail/Brushed-Flat-Aluminium-7075-T6-Sheets_1600743210085.html?spm=a2700.galleryofferlist.normal_offer.d_title.179113a0uTPDNV&priceId=6d49a9bcabca4170b16f92ea9d659b09.

29. PTFE. https://www.alibaba.com/product-detail/PTFE-Film-0-05mm-0-1mm_1601221455351.html?spm=a2700.galleryofferlist.topad_classic.d_title.303e13a0lEySLO&priceId=76d8216fdc85455f9a8b2ee2e98c88a6.

30. Vlǎdoiu, I., Stafe, M., Neguţu, C. & Popescu, I. M. The dependence of the ablation rate of metals on nanosecond laser fluence and wavelength. *UPB Scientific Bulletin, Series A: Applied Mathematics and Physics* **70**, 119–126 (2008).

31. Igarashi, K., Iwasa, M., Sasaki, T., Takahashi, K. & Kikuchi, T. Performance of laser ablation propulsion with a high-repetition rate and high-power laser. *AIP Adv.* **12**, (2022).

32. Li, S. S. *et al.* Development and applications of aluminum alloys for aerospace industry. *Journal of Materials Research and Technology* **27**, 944–983 (2023).

33. Serafetinides, A. A., Makropoulou, M. I., Skordoulis, C. D. & Kar, A. K. Ultra-short pulsed laser ablation of polymers. *Appl. Surf. Sci.* **180**, 42–56 (2001).

34. Sinko, J. E. & Gregory, D. A. CO2 laser ablation impulse generation with polymer propellants. *J. Propuls. Power* **27**, 1121–1130 (2011).

35. Dhanumalayan, E. & Joshi, G. M. Performance properties and applications of polytetrafluoroethylene (PTFE)—a review. *Adv. Compos. Hybrid Mater.* **1**, 247–268 (2018).

36. Zheng, Z. Y. *et al.* Characteristic investigation of ablative laser propulsion driven by nanosecond laser pulses. *Appl. Phys. A Mater. Sci. Process.* **83**, 329–332 (2006).

37. Pakhomov, A. V., Thompson, M. S., Swift, W. & Gregory, D. A. Ablative laser propulsion: Specific impulse and thrust derived from force measurements. *AIAA Journal* **40**, 2305–2311 (2002).

38. Jamil, Y. *et al.* Measurement of ablative laser propulsion parameters for aluminum, Co-Ni ferrite and polyurethane polymer. *Appl. Phys. A Mater. Sci. Process.* **110**, 207–210 (2013).

39. Rao, S. *et al.* MOFs for Ultrahigh Efficiency Pulsed Laser Micropropulsion. *Advanced Materials* **2306228**, 1–10 (2024).

40. Rao, S. *et al.* Optical-Propulsion Metastructures. *Advanced Materials* **36**, 2406384 (2024).

41. Phipps, C. R., Luke, J. R., McDuff, G. G. & Lippert, T. Laser-driven micro-rocket. *Appl. Phys. A Mater. Sci. Process.* **77**, 193–201 (2003).

42. Suzuki, K., Sawada, K., Takaya, R. & Sasoh, A. Ablative impulse characteristics of polyacetal with repetitive CO 2 laser pulses. *J. Propuls. Power* **24**, 834–841 (2008).

43. Lippert, T. *et al.* Novel applications for laser ablation of photopolymers. *Appl. Surf. Sci.* **186**, 14–23 (2002).

44. Zhang, Y. *et al.* Laser propulsion with a high specific impulse using a thin film propellant. *Chinese Physics B* **20**, (2011).

45. Battocchio, P. *et al.* Improved laser ablation propulsion efficiency in composite polymers, containing reduced graphene oxide, by the spontaneous formation of a confining layer. *Appl. Surf. Sci.* **687**, 162251 (2025).

46. Phipps, C. R., Llc, P. A. & Luke, J. Laser ablation of organic coatings as a basis for micropropulsion. https://doi.org/10.1016/j.tsf.2003.11.138 (2017) doi:10.1016/j.tsf.2003.11.138.

47. Wang, L., Tam, W. Y., Zhao, Q. & Wang, X. Quantitative measurement and mechanism analysis of the high-efficiency laser propulsion of a graphene sponge. *Opt. Express* **28**, 33869 (2020).

48. Gaudenzi, R., Stefani, D. & Cartamil-Bueno, S. J. Light-induced propulsion of graphene-on-grid sails in microgravity. *Acta Astronaut.* **174**, 204–210 (2020).

49. Wang, L., Dai, M., Zhao, Q. & Wang, X. Mechanism research of the laser propulsion of bulk graphene sponge material through high vacuum experiment. *Vacuum* **191**, 110334 (2021).

50. Wang, L., Wang, S., Zhao, Q. & Wang, X. Macroscopic laser pulling based on the Knudsen force in rarefied gas. **31**, 2665–2674 (2023).

51. Khattab, O. *et al.* Photon-assisted displacement of directionally freeze-dried symmetric graphene aerogels. *Mater. Des.* **260**, 114955 (2025).
